# *Spink2* Modulates Apoptotic Susceptibility and Is a Candidate Gene in the *Rgcs1* QTL That Affects Retinal Ganglion Cell Death after Optic Nerve Damage

**DOI:** 10.1371/journal.pone.0093564

**Published:** 2014-04-03

**Authors:** Joel A. Dietz, Margaret E. Maes, Shuang Huang, Brian S. Yandell, Cassandra L. Schlamp, Angela D. Montgomery, R. Rand Allingham, Michael A. Hauser, Robert W. Nickells

**Affiliations:** 1 Department of Ophthalmology and Visual Sciences, University of Wisconsin, Madison, Wisconsin, United States of America; 2 Department of Biostatistics, University of Wisconsin, Madison, Wisconsin, United States of America; 3 Center for Human Genetics, Department of Medicine, Duke University Medical Center, Durham, North Carolina, United States of America; Casey Eye Institute, United States of America

## Abstract

The *Rgcs1* quantitative trait locus, on mouse chromosome 5, influences susceptibility of retinal ganglion cells to acute damage of the optic nerve. Normally resistant mice (DBA/2J) congenic for the susceptible allele from BALB/cByJ mice exhibit susceptibility to ganglion cells, not only in acute optic nerve crush, but also to chronic inherited glaucoma that is characteristic of the DBA/2J strain as they age. SNP mapping of this QTL has narrowed the region of interest to 1 Mb. In this region, a single gene (*Spink2*) is the most likely candidate for this effect. *Spink2* is expressed in retinal ganglion cells and is increased after optic nerve damage. This gene is also polymorphic between resistant and susceptible strains, containing a single conserved amino acid change (threonine to serine) and a 220 bp deletion in intron 1 that may quantitatively alter endogenous expression levels between strains. Overexpression of the different variants of *Spink2* in D407 tissue culture cells also increases their susceptibility to the apoptosis-inducing agent staurosporine in a manner consistent with the differential susceptibility between the DBA/2J and BALB/cByJ strains.

## Introduction

Glaucoma is a complex genetic disease that is characterized by the degeneration of the optic nerve and the apoptotic death of retinal ganglion cells [1], [2]. Although several genetic loci, and some genes, have been identified that affect the onset and severity of glaucoma, these have mostly been limited to rare forms of the disease in which pedigrees of individuals with clear inheritance patterns are apparent or account for a small percentage (∼5%) of Primary Open Angle Glaucoma (POAG), the major form of glaucoma [3]. To address the complex genetic nature of POAG, several large multi-center genome-wide association studies (GWAS) of POAG have been conducted. These studies have identified at least three regions of interest; *CDKN2BAS*, *SIX6*, and chromosome 8q22 [4], [5], [6]. Although important, it is suspected that the number of associations will increase as the size and power of various glaucoma datasets grow [7]. A potential difficulty in elucidating the genetics of glaucoma likely lies in the heterogeneity of diseases that are commonly defined as “glaucoma”. Thus, stratification of data sets is complicated until more precise phenotype classifications can be defined.

With these limitations in mind, we attempted an alternative approach to identify genetic loci that could influence the onset and progression of glaucoma by evaluating the effect of genetic background in mice in response to a common insult meant to mimic glaucomatous damage. The typical pattern of ganglion cell death in the retina observed in glaucomatous optic neuropathy that is caused by elevated intraocular pressure (IOP), is produced by axonal damage at the level of the optic nerve head [8], [9]. A model of axonal damage in glaucoma is optic nerve crush. We monitored the effect of this controlled mechanical insult to the optic nerve on mice from 15 different inbred genetic backgrounds. This experiment led to the identification of resistant (DBA/2J) and susceptible (BALB/cByJ) parental lines [10]. Subsequent reciprocal backcross studies, followed by genetic screening of a large mapping population, resulted in the description of a 25 cM interval on chromosome 5 (Chr5.loc34-59 cM comprising 58 Mb) that was dominant for the resistant phenotype [11]. This QTL was designated Retinal ganglion cell susceptible 1 (*Rgcs1*).

Here, we show that congenic breeding of the BALB/c *Rgcs1* locus onto the resistant DBA/2J genetic background (creating the substrain DBA/2J.BALB*^Rgcs1^*) confers increased susceptibility to acute optic nerve damage. DBA/2J mice also develop chronic elevated intraocular pressure (IOP) and glaucomatous optic nerve damage [8], [12], [13], [14], [15]. Age-matched DBA/2J.BALB*^Rgcs1^* substrain animals exhibit similar kinetics of IOP elevation, but a more severe glaucomatous phenotype. Further mapping of the *Rgcs1* locus using single nucleotide polymorphisms (SNPs) narrowed the region of interest to approximately 1 Mbp, containing 23 known genes. One of these genes, Serine protease inhibitor Kazal type 2 (*Spink2*) is expressed in retinal ganglion cells and is dramatically up-regulated after optic nerve crush in mice. In vitro analysis of *Spink2* overexpression indicates that it can modulate the susceptibility of cells to apoptotic stimuli.

## Results

### The BALB/cByJ *Rgcs1* Region Confers Susceptibility on the Resistant DBA/2J Genetic Background

**Figure 1:**
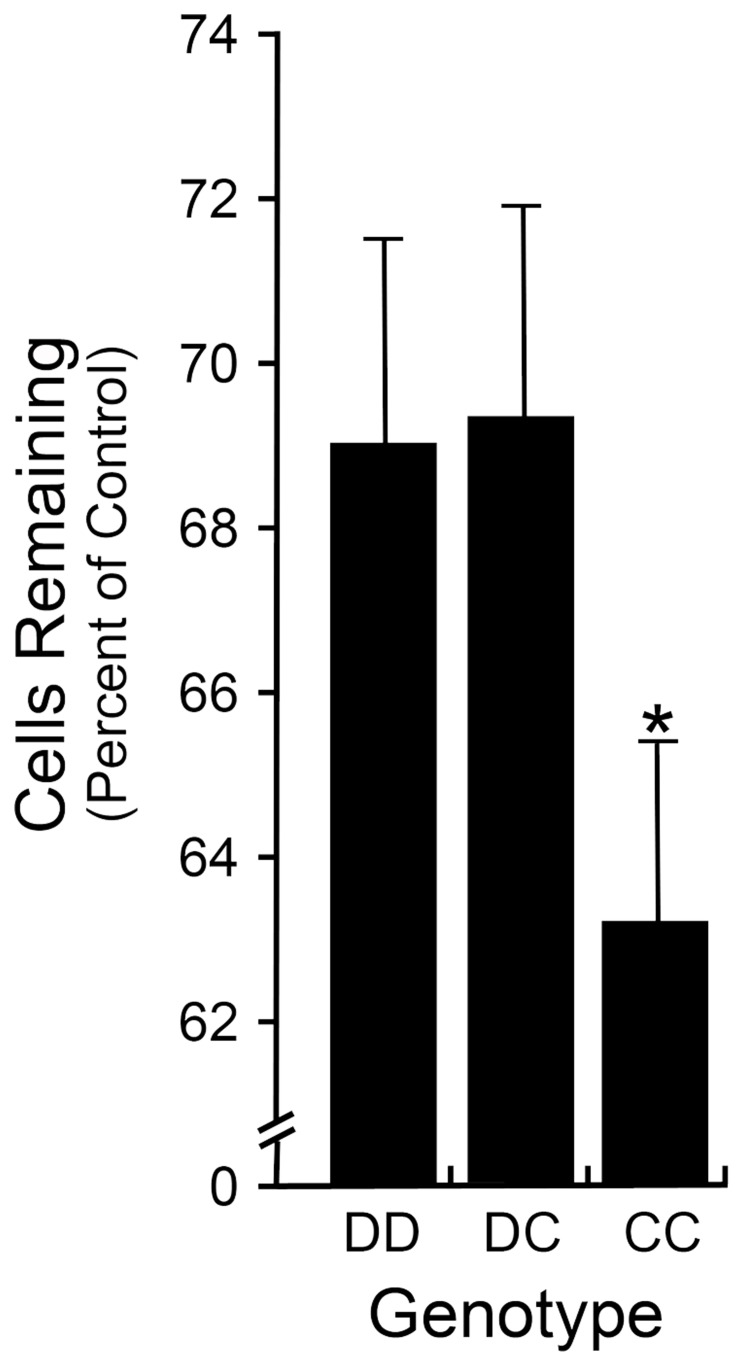
DBA/2J.BALB*^Rgcs1^* substrain mice exhibit a susceptible phenotype after optic nerve crush. Bar graph showing cell loss after optic nerve crush (mean ± sem) in DBA/2J mice carrying the BALB/cByJ *Rgcs1* allele (C). Mice with the CC allele exhibit a significantly greater percentage of cell loss in the ganglion cell layer than either DD or DC mice (P = 0.024, CC compared to the DD and DC animals combined). This difference in phenotype mimics the difference between purebred DBA/2J mice compared to purebred BALB/cByJ mice [10]. A minimum of 20 mice was assayed per group.

The *Rgcs1* QTL from BALB/cByJ mice was identified as a recessive allele that was linked to greater cell loss after optic nerve crush in these mice. To test further the ability of this region to modulate the cell death phenotype in the resistant DBA/2J strain, the region of chromosome 5 flanked by microsatellite markers D5Mit254 (34 cM) and D5Mit338 (59 cM) was bred onto the DBA/2J background through 10 successive generations. Substrain mice, heterozygous and homozygous for the BALB/cByJ locus were then subjected to the optic nerve crush procedure at 8 weeks of age and compared to pure bred DBA/2J animals. DBA/2J mice exhibit retinal ganglion cell loss after optic nerve damage [1], but the rate and amount of cell loss is less than other strains [10], which is why they were classified as the “resistant” strain. Consistent with previous studies, mice homozygous for the BALB/cByJ *Rgcs1* region exhibited significantly more cell loss (to levels similar to pure bred BALB/cByJ mice – data not shown) compared to mice carrying the DBA/2J allele (P = 0.024, Figure 1).

**Figure 2:**
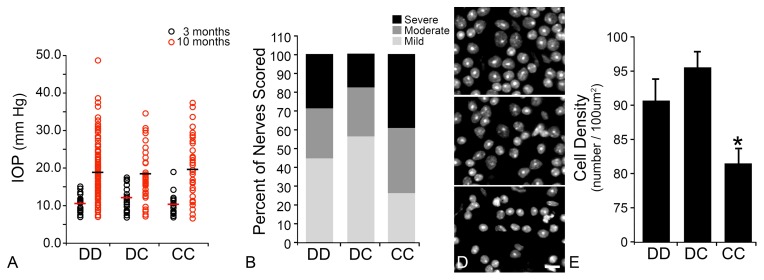
DBA/2J.BALB*^Rgcs1^* substrain mice have more severe glaucomatous damage. (A) Intraocular pressures (IOP) of eyes from 3 month and 10 month DBA/2J wild type and substrain mice. Mice homozygous for the BALB/cByJ allele of *Rgcs1* (CC) exhibit no difference in IOPs at either age (P = 0.275, 3 months; P = 0.889, 10 months) compared to mice carrying at least one DBA/2J allele for *Rgcs1* (DD and DC, respectively). For CC mice data was collected from 15 and 42 eyes at 3 months and 10 months, respectively. For DC mice, data was collected from 28 and 41 eyes, respectively. For DD mice, data was collected from 20 and 136 eyes, respectively. (B) Bar graph showing the frequency of mild, moderate, and severe optic nerve damage in 10 month old DBA/2J mice carrying either the D or C alleles for *Rgcs1*. Mice homozygous for the BALB/c allele (CC) have a higher frequency of severe optic nerve damage than mice with the D allele (β^2^ P = 0.013, CC compared to the combined DD and DC animals). Nerves from 163, 90, and 40 DD, DC, and CC mice, respectively were scored. (C) Images of whole mounted retinas, surface stained with DAPI showing exemplars of mild, moderate, and severe damage. (D) Bar graph showing the mean retinal cell density of 10 month old DBA/2J mice. (*P<0.001, CC compared to DD and DC samples combined).

DBA/2J mice also develop anterior chamber abnormalities leading to pathology of the trabecular meshwork and ocular hypertension [13], [16]. By 8 months of age, a majority of these animals exhibit elevated IOP and, by 10 months, glaucomatous pathology of the retina and optic nerve [14], [15]. DBA/2J mice congenic for the BALB/cByJ *Rgcs1* allele (DBA/2J.BALB*^Rgcs1^*) developed increased IOP with age, equivalent to IOP changes in DBA/2J animals (P = 0.889, Figure 2A). These mice, however, had more severe glaucomatous damage to both the optic nerve and retina, compared with pure bred and heterozygous age-matched mice (P = 0.013 for optic nerves and P<0.001 for retinas, Figure 2). These results support the hypothesis that the *Rgcs1* locus can modify the level of glaucomatous damage in a manner consistent with the acute optic nerve damage paradigm.

### SNP Mapping of the *Rgcs1* QTL

**Figure 3:**
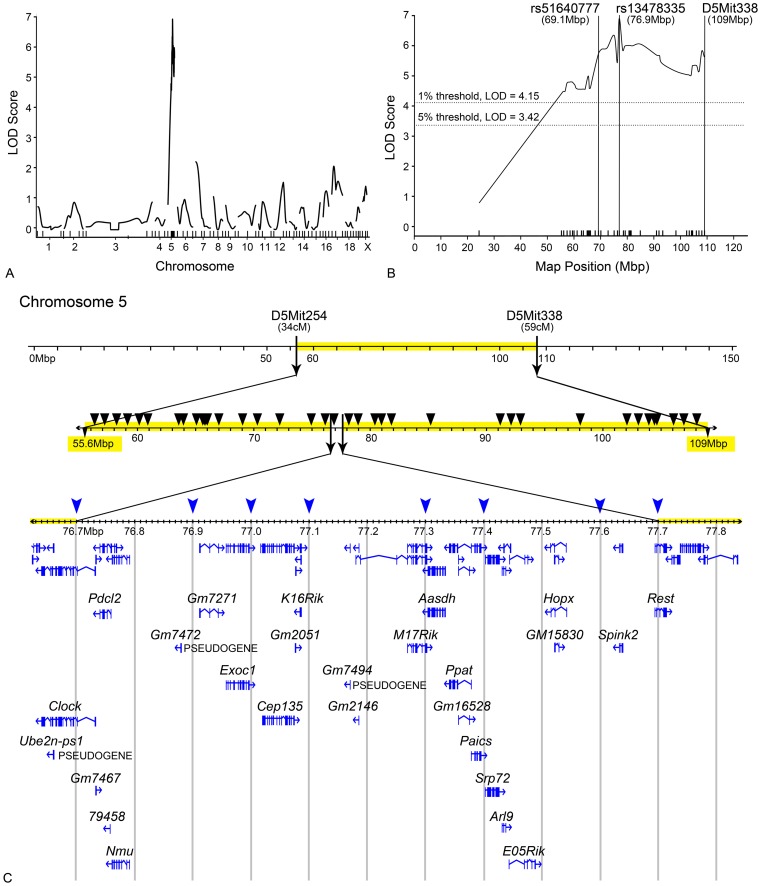
Narrowing of the *Rgcs1* QTL to 1 Mb by SNP analysis. (A) A genome wide scanone of LOD scores for 252 mice generated by the F2 generation of a DBA/2J and BALB/cByJ cross. Data obtained from genome-wide microsatellite markers combined with 38 SNPs spaced throughout the 58 Mb region of chromosome 5 between Chr5:loc34-59 cM. A single significant peak with a LOD score of 6.81 was detected. (B) Detail of the scanone peak on chromosome 5 showing the highest association with SNP rs13478335. (C) Genomic map of mouse chromosome 5. The region highlighted in yellow indicates the size of the *Rgcs1* QTL as defined by microsatellite markers in Dietz et al [11]. Black arrowheads indicate the positions of the first screen using SNPs across this region. Blue arrowheads indicate the position of SNPs between 76.7 and 77.7 Mb used in a second round of screening. No further narrowing of the interval was achieved in this second round. Known genes and putative coding regions (ENSEMBL genome browser) in this interval are shown.

SNP analysis using 37 informative SNP markers, all within the region of the original limits of the *Rgcs1* QTL, were conducted on 252 mice generated in an F2 cross (see Methods). This analysis defined further a significant association with a peak LOD score of 6.81 centered between 76.7 and 77.7 Mb (Figure 3), well above the estimated 99% confidence interval estimate of 4.15. A second round of mapping using 8 additional SNPs situated within this 1 Mb region confirmed this peak LOD score. LOD scores increased for all markers after normal transformation of the mapping population data, but did not change the position of peak association (data not shown). The fitted LOD curve on chromosome 5 using high resolution mapping with SNPs inside the 1 Mb region also yielded a potential bimodal structure suggestive of 2 QTLs in this region. This possibility was not supported by a “scantwo” analysis based on this assumption, however. Figure S1 shows the dominant inheritance pattern of the resistant phenotype as a function of the rs13478335 SNP. F2 animals, homozygous for the BALB/cByJ allele (CC), had significantly more cell loss (P<0.001) than mice either homozygous or heterozygous for the DBA/2J allele (DD and DC). Overall, this allele accounts for ∼11% of the cell death phenotype after optic nerve crush. This is consistent with the level of increased susceptibility in DBA/2J.BALB*^Rgcs1^* (CC) mice to optic nerve crush (Figure 1) and previous estimates calculated from the data obtained using microsatellite screening of a subset of this mapping population [11].

### Optic Nerve Crush Mediated Expression Changes of Genes in the *Rgcs1* Region

The 1 Mbp region of *Rgcs1* identified by SNP mapping contains 23 open reading frames identified as genes and 3 pseudogenes (see Figure 3A). Strain comparison analysis of this region using the mouse genomes database (Wellcome Trust Sanger Institute) revealed two potentially interesting structural changes in *Aasdh* and *Spink2* that distinguished the two strains. *Aasdh* contains a 20 bp deletion in intron 15 of the DBA/2J allele, which is predicted to affect splicing of the primary transcript. RT-PCR from exon 14 to 16, however, did not reveal any splice variants of this mRNA in DBA/2J or BALB/cByJ retinal samples (data not shown). In addition, a 220 bp deletion was identified in intron 1 of the *Spink2* gene of BALB/cByJ mice, which could affect expression levels (see below).

**Figure 4:**
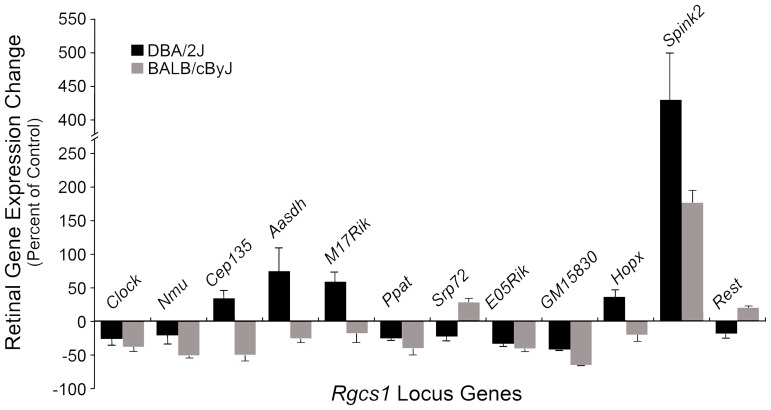
Expression changes of genes in the Rgcs1 locus after optic nerve crush. Primers for all the genes present in the peak 1*Rgcs1* QTL (Table S1) were used to amplify cDNAs from the optic nerves and retinas of DBA/2J and BALB/cByJ mice after optic nerve crush. Genes that showed expression in control eyes were then examined by real time qPCR. Data shown represents the percentage of expression in experimental samples relative to fellow unoperated samples. In the retina only *Spink2* showed a substantial and significant (P) increase in both strains after crush. Changes in transcript abundance in the optic nerves after crush are shown in Figure S2.

To evaluate further the potential importance of these genes in the optic nerve crush phenotype, we also conducted expression analyses of them in both the retina and optic nerve after crush. Multiple primer sets corresponding to exon sequences of the putative genes were synthesized and regular RT-PCR was performed on cDNA synthesized from total RNA isolated from both retinas and optic nerves of DBA/2J and BALB/cByJ mice. Of the 23 putative genes in the area, we could amplify cDNA for 12 genes in either or both tissues. Quantitative PCR analysis was then performed on samples of control or experimental eyes, 7 days after optic nerve crush. In the optic nerve samples, only *Cep135* was found to be consistently upregulated in damaged nerves (Figure S2). In the retina, only *Spink2* was found to be noticeably upregulated after optic nerve crush (Figure 4). Absolute levels of *Spink2* mRNAs (Figure S3) indicated that BALB/cByJ mice expressed higher levels of this gene, both before and after optic nerve crush. Similarly, western blot analyses of SPINK2 levels in DBA/2J and DBA/2J.BALB*^Rgcs1^* mice showed higher levels of protein in the retinas of animals carrying the BALB/cByJ allele. Optic nerve crush stimulated variable increases in SPINK2 protein (ranging from 25–300%) after optic nerve crush in both strains (Figure S3).

### *Spink2* is Expressed in the Mouse Retina

Although *Cep135* is upregulated in optic nerves after damage, this gene encodes a protein that is involved in centriole formation and microtubule organization [17]. Therefore, it is likely that its upregulation after optic nerve damage reflects the proliferation of glial cells in the region of the lesion reported by others [8], [18]. Since this increase in expression was expected for *Cep135*, we have not focused further investigation on it at this time.

**Figure 5:**
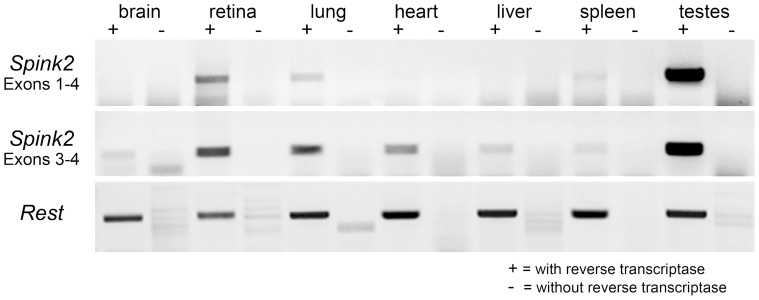
Tissue distribution of *Spink2* expression in the mouse. Reverse transcriptase-PCR analysis of *Spink2* transcript presence in different mouse tissues. Previous reports have indicated that *Spink2* is only expressed in the mouse testes [19]. By comparing RT-PCR reactions using both long (exon 1–4) and short (exon 3–4) amplimers, a transcript for *Spink2* was detected in a variety of tissues, suggesting that this gene is ubiquitously transcribed, albeit at levels that appear lower than expression in the testes. Of note, retina appears to have relatively high levels of expression compared to brain tissue.

Alternatively, *Spink2* expression was not expected after optic nerve crush. This gene is highly expressed in the testes where it plays a role in spermatogenesis [19]. The human homolog, *SPINK2*, is also expressed in some leukemia cells lines [20]. To our knowledge, this is the first report of *Spink2* expression in the CNS. To examine the distribution of *Spink2* expression in mouse tissues, we conducted RT-PCR amplification with 2 separate primer pairs for this cDNA. Figure 5 shows the relatively robust presence of *Spink2* transcript in the retina using primers spanning exons 1–4, and lesser amounts in all other tissues. *Spink2* mRNA in these latter tissues was only detected by amplification of the smaller 242 bp fragment, spanning exons 3–4. This may reflect more efficient PCR of a smaller fragment, which could better detect low abundant transcripts. Conversely, it may indicate that other tissues express a predicted alternate splice variant of *Spink2* mRNA, which eliminates exon 1 in favor of part of the first intron (ENSEMBL). The most abundant source of *Spink2* appeared to be testes, consistent with other observations.

**Figure 6:**
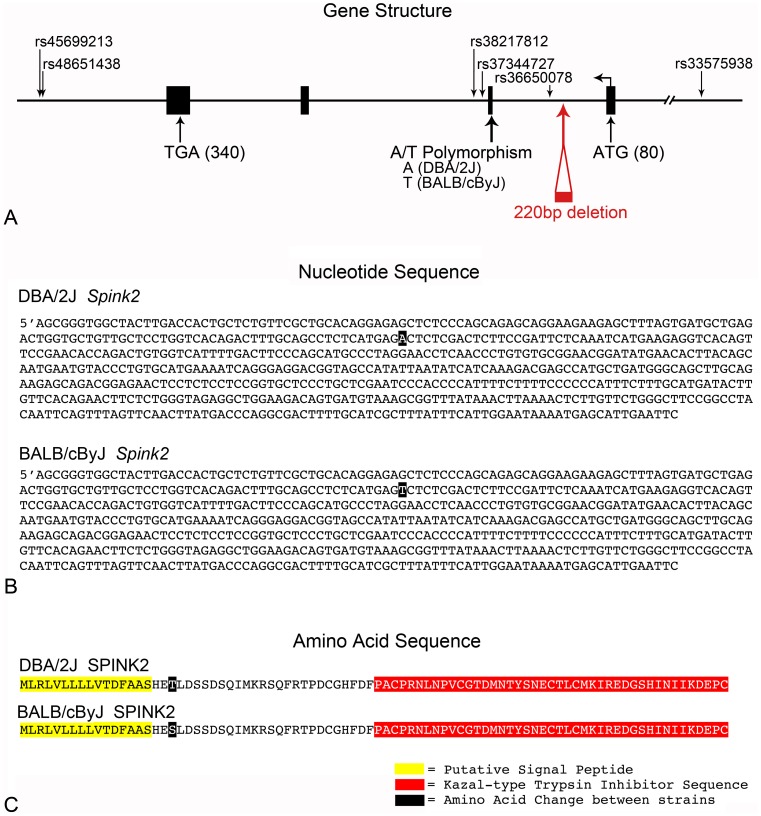
DBA/2J and BALB/cByJ mice express variants of *Spink2*. (A) Gene structure for mouse *Spink2*. Exons are indicated by large filled boxes and the gene is transcribed from right to left (bent arrow). The start (ATG) and the stop (TGA) codons are shown. The positions of known SNPs between the two parental strains (Mouse Genome Informatics database, Jackson Laboratories) are shown. Sequence analysis of exons revealed an A/T SNP in exon 2, which was also identified in the Mouse Genomes Project (Wellcome Trust Sanger Institute), although we detect the A allele in DBA/2J mice, not BALB/cByJ. Additionally, BALB/c mice have a reported 220 bp deletion in intron 1. (B) Sequence of the mRNA for Spink2 in the 2 parental strains showing the position of the A/T polymorphism. (C) Amino acid sequence and structure of the SPINK2 protein. The A/T SNP changes amino acid 19 from a threonine (DBA/2J) to a serine (BALB/cByJ). This change lies outside of a putative signal peptide (highlighted in yellow) and the Kazal protease inhibitor domain (highlighted in red).

Sequence analysis of multiple *Spink2* cDNA clones isolated from DBA/2J and BALB/cByJ mice revealed a single A/T polymorphism in exon 2. This polymorphism was confirmed by sequencing exon 2 from the genomic DNA of 5 separate mice from each strain. The nucleotide difference results in a conserved amino acid change (Threonine^DBA/2J^ to Serine^BALB/cByJ^) at position 19. This change did not occur in either the putative signal peptide or in the Kazal-type protease inhibitor domain (Figure 6). This polymorphism maps to a SNP reported in the Mouse Genomes Project database of the Wellcome Trust Sanger Institute (rs241800406), with the exception that we observe the A variant in DBA/2J mice rather than BALB/cByJ (T variant). Mice carrying the A variant also genotype as DBA/2J using SNPs situated on either side of this position (data not shown). Since *Spink2* is in the reverse orientation on chromosome 5, this may be a reference strand issue. In addition to the SNP in the *Spink2* coding region, analysis of this locus using the Mouse Genome Informatics database at Jackson Laboratories revealed 4 other non-coding SNP differences within 15 kb on either side of the gene. Two of these are situated in the second intron, close to the 3′ end of exon 2, one is centered in the first intron, and one is located 6924 bp upstream of the putative transcription start site (Figure 6). SNPs and/or the intronic deletion located in non-coding regions of the *Spink2* gene may occur in regulatory regions and could account for the increased levels of transcript observed in BALB/cByJ mice (Figure S3). Alternatively, this region may be subject to copy number variations (CNVs) between strains. A literature search of CNVs in different mouse strains [21] showed no reported gains or deletions between these mice within the 1 Mbp region defining the *Rgcs1* locus. To confirm this we conducted qPCR copy number assays on genomic DNA specific for exon 4 of the *Spink2* gene. These assays predicted only 2 alleles for *Spink2* in both DBA/2J and BALB/cByJ strains (data not shown).

**Figure 7:**
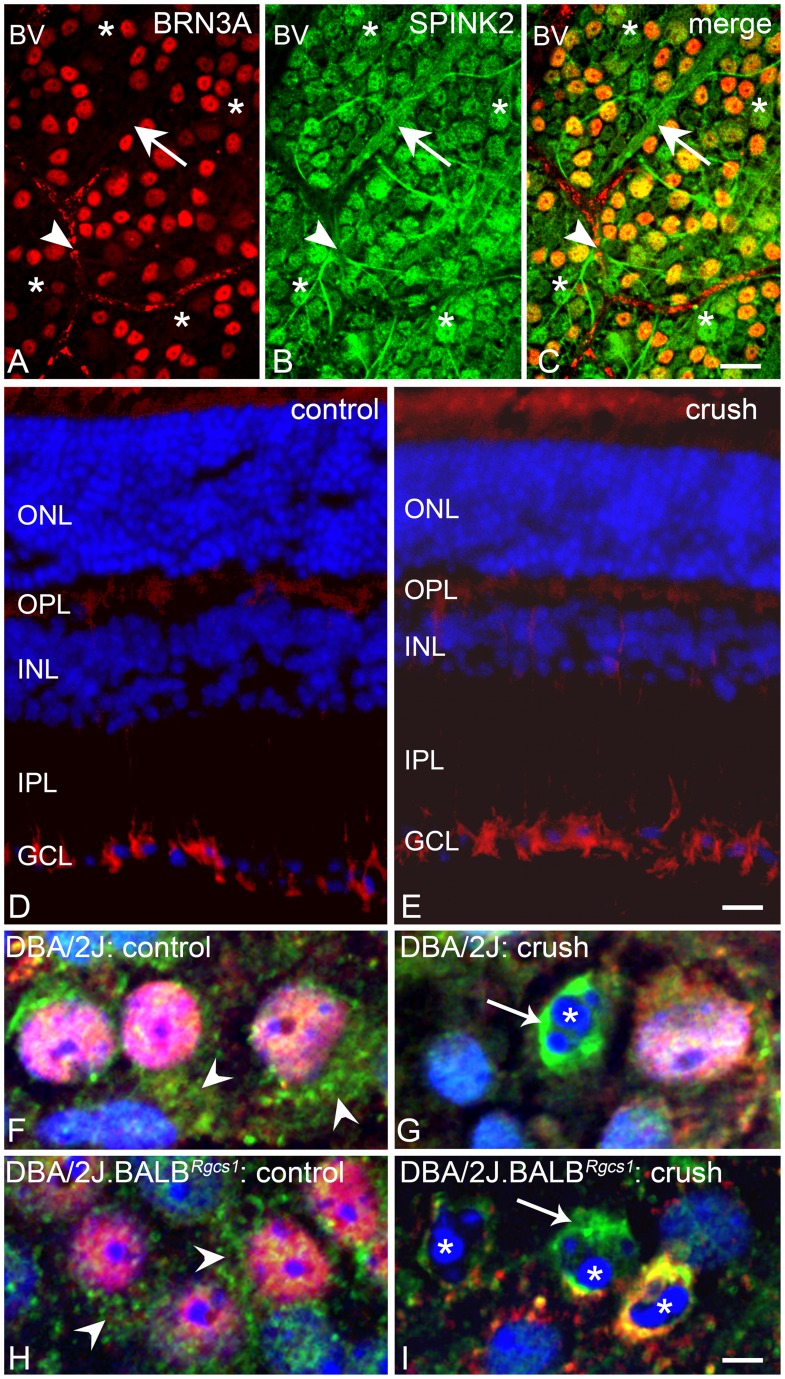
SPINK2 protein is localized to cells in the ganglion cell layer. (A–C) Retinal whole mounts stained for the ganglion cell marker BRN3A (A) and SPINK2 (Santa Cruz goat polyclonal) (B). A majority of BRN3A positive cells are positive for SPINK2 staining. Additionally, nerve fiber bundles emanating from ganglion cells are SPINK2 positive (arrow). SPINK2 also stains BRN3A negative cells (asterisks), which may be displaced amacrine cells or some of the 10–15% of ganglion cells that do not stain for this transcription factor [23]. SPINK2 also stains cells with morphology typical of astrocytes (arrowhead). BV = blood vessel. Size bar = 30 μm. (D,E) Frozen sections of a control (D) and crush (E) mouse retina (DBA/2J.BALB*^Rgcs1^* mouse shown) stained for an antibody against the C-terminus of mouse SPINK2 (ProSci Inc., rabbit polyclonal). This antibody predominantly stains the cells in the ganglion cell layer (GCL), although some light staining is evident in the innermost cells of the inner nuclear layer (INL) and the inner plexiform layer (IPL) and outer plexiform layer (OPL). This antibody may also stain putative Müller cell processes (see Figure S5). There is no appreciable change in the staining pattern of SPINK2 before and after crush when viewed on sections. The overall pattern of staining shown here is consistent among 3 different SPINK2 antibodies tested. Size bar = 50 μm. (F–I) Retinal whole mounts from DBA/2J (F,G) and DBA/2J.BALB*^Rgcs1^* (H,I). Ganglion cells positive for BRN3A (yielding pink colored nuclei with the DAPI counterstain – arrowheads) show diffuse SPINK2 staining (green, rabbit polyclonal, see ref 18) in control retinas (F,H). At 7 days after optic nerve crush, some cells in the retinas of both strains exhibit intense SPINK2 immunoreactivity (examples marked by arrows) that surrounded nuclei with condensed and fragmented chromatin (asterisks). At this stage, ganglion cells exhibit only limited staining for BRN3A (see text). Size bar = 5 μm.

We also examined the localization of SPINK2 protein in the DBA/2J mouse retina using immunofluorescence. Staining of sections with 3 different antibodies against SPINK2 (see Methods) yielded similar localization patterns in which the strongest staining was detected in cells of the ganglion cell layer, along with weaker staining in the inner nuclear layer (Figure 7). There were also some differences in the localization patterns of the 3 antibodies. The rabbit polyclonal antibody from ProSci (see Methods) also revealed staining in putative Müller cell processes (Figure S4), while a rabbit polyclonal used in previous studies [19], exhibited strong immunoreactivity in the nerve fibers of the ganglion cell layer. Expression of SPINK2 in retinal ganglion cells was confirmed by colocalization with BRN3A, a class IV POU-domain containing transcription factor that is selectively expressed by approximately 85–90% of the ganglion cells in the rodent retina [22], [23]. Confocal images of retinal whole mounts indicated SPINK2 also stained ganglion cell nerve fibers. These images showed, however, that SPINK2 was not restricted to ganglion cells, but was also expressed in cells resembling astrocytes and cells not positive for BRN3A (Figure 7). In sections, no dramatic change in SPINK2 distribution was observed in retinas after optic nerve crush in both DBA/2J and DBA/2J.BALB*^Rgcs1^* mice (Figure 7). Staining of retinal whole mounts, however, showed that cells scattered throughout the crushed retinas exhibited very intense SPINK2 immunoreactivity. These cells were almost exclusively associated with nuclei containing condensed and fragmented chromatin (Figure 7) and were more prevalent in the DBA/2J.BALB*^Rgcs1^* substrain. Although these intensely labeled cells were not always associated with BRN3A staining, we presumed that they were ganglion cells in later stages of apoptosis, when *Brn3a* expression is known to be significantly down-regulated [24], [25].

### Exogenous *Spink2* Expression Increases Susceptibility to an Apoptotic Stimulus

**Figure 8:**
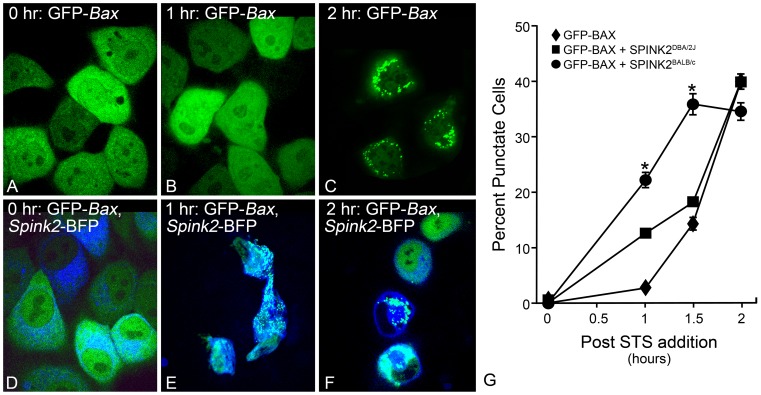
Overexpression of *Spink2* increases susceptibility to an apoptotic stimulus in D407 cells. The *Spink2* coding regions from DBA/2J and BALB/cByJ mice were cloned in frame with blue fluorescent protein (BFP) and co-nucleofected with a GFP-*Bax* reporter plasmid into D407 cells. Apoptosis was induced with staurosporine (STS) and the rate of GFP-BAX aggregation was monitored. (A–C) D407 cells nucleofected with GFP-BAX only. (A) Cells 24 hours after nucleofection showing diffuse distribution of the GFP-BAX fusion protein. (B) One hour after addition of STS, GFP-BAX remains diffusely localized. (C) By 2 hours after addition of STS, GFP-BAX has aggregated into puncta, consistent with its activation and translocation to the mitochondria during apoptosis [48], [49]. (D–F) D407 cells co-nucleofected with GFP-*Bax* and the BALB/cByJ *Spink2*-BFP fusion plasmid. The presence of SPINK2 accelerates the apoptotic response of D407 cells to STS as a function of GFP-BAX aggregation. Size bar = 10 μm. (G) Graph showing the rate of cells with aggregating BAX after STS addition. The BALB/cByJ variant of *Spink2*-BFP yields the fastest rate of aggregation, while the DBA/2J variant yields an intermediate response. Data shown is the mean (± SD) of 3–5 independent experiments. (*P<0.0001 relative to both DBA/2J *Spink2*-BFP and GFP-BAX groups).

We examined if overexpression of exogenous *Spink2* could increase the susceptibility of tissue culture cells to an apoptotic stimulus. Plasmids were constructed that created fusion proteins between the complete coding region of *Spink2* from either DBA/2J or BALB/cByJ mice, fused to a blue fluorescent protein (BFP) tag. This fusion protein performed similarly to constructs expressing SPINK2 protein alone, indicating no change in activity caused by the BFP addition (data not shown). We then used a BAX aggregation assay to evaluate the activation of apoptosis in D407 cells challenged with staurosporine (STS). These cells were chosen because they exhibit intrinsic apoptosis after an STS challenge, and retain excellent morphology in live-cell imaging experiments. In this assay, cells were transfected with either a GFP-*Bax* expression construct, or GFP-*Bax* and *Spink2*-BFP constructs together. After 24 hours, the cells were challenged with 1 μM STS and followed by live-cell imaging for 2 hours. STS activates GFP-BAX aggregation in D407 cells within 1.5 to 2 hours after incubation (Figure 8). Cells expressing either variant of the *Spink2*-BFP plasmid, however, exhibited an accelerated response, with a significantly greater proportion of cells with GFP-BAX aggregation at 0.5 and 1.0 hours after STS challenge (P<0.001). Consistent with an increase in susceptibility in BALB/cByJ mice, the greatest increase in susceptibility of D407 cells to STS was observed in cells expressing the BALB/cByJ variant of *Spink2* (P<0.0001). In parallel experiments, overexpression of the *Hopx* gene from both strains had no effect on increasing D407 cell susceptibility (data not shown). These data support the hypothesis that SPINK2 protein function is affected by the amino acid change at position 19. In separate experiments, D407 cells were transfected with either a *Spink2* ^BALB/cByJ^ expressing plasmid, or a plasmid expressing GFP as a transfection control. Cell death was assessed using a combined caspase 3/7 activity assay. At 8 hours post STS addition, *Spink2* transfected cells demonstrated greater caspase activation than GFP-transfected cells, or non-transfected cells (Figure S5), corroborating the data obtained using the BAX aggregation assay.

## Discussion

### *Rgcs1* Susceptibility and Glaucoma

The underlying principal of the approach of screening inbred mice to identify loci that affect susceptibility of retinal ganglion cells to optic nerve damage was to help catalog genes that might contribute to the complex glaucoma phenotype. An important step in this process was to confirm that loci that affect optic nerve crush (an acute damage paradigm) would also affect cell death in glaucomatous damage (a chronic paradigm). We took advantage of the fact that the DBA/2J line, the resistant strain in the original screen [10], is also a well-established mouse model of chronic inherited glaucoma [12], [14], [15], [16]. DBA/2J.BALB*^Rgcs1^* substrain mice exhibit a BALB/cByJ phenotype in the acute optic nerve crush protocol at an age of 8–10 weeks, well before the onset of the glaucomatous phenotypes attributed to the DBA/2J strain. These mice exhibit iris atrophy and pigment dispersion, leading to elevated IOP along the same time course as pure bred DBA/2J mice. Evaluation of two metrics of glaucomatous damage, optic nerve degeneration and loss of retinal ganglion cell somas, shows that substrain animals have a statistically greater level of damage as compared to both pure bred and heterozygous substrain mice. This demonstrates that the *Rgcs1* allele affects the damage pathways activated in both the acute and chronic models of optic nerve damage.

The syntenic region of the *Rgcs1* QTL lies between 56.3 and 57.9 Mbp of human chromosome 4. To date no significant associations with genes in this region, including *SPINK2*, have been made with glaucomatous pathology in either of the NEIGHBOR or GLAUGEN GWAS studies. There are multiple reasons for a lack of association, including insufficient power of the human data sets to find an association with a gene that has only a partial contributory role to glaucomatous pathology. Most important, however, this underscores a potential limitation in studies involving human glaucoma subjects. The criteria for inclusion of patient samples in the affected population included clinical indicators such as optic nerve head appearance, along with thinning of the nerve fiber layer, elevated IOP, and/or defects on a visual field exam [6]. While inclusion required that multiple of these parameters were met, they were likely less precise quantitatively, and different, than the quantitative trait (ganglion cell soma loss) used to identify the *Rgcs1* locus. To date, phenotyping in all glaucoma genetic analyses has been based on essentially a binary model, presence or absence of clinically defined glaucoma within predefined age limits, such as POAG versus primary congenital glaucoma. It is challenging to determine genetic influences that have subtle effects on susceptibility to disease, which requires long periods of clinical observation to measure. Further stratification of disease severity based on the rate of progression associated with specific IOP profiles may be required to identify associations for genes like *SPINK2*. For example, even though DBA/2J.BALB*^Rgcs1^* mice develop a more severe glaucoma phenotype than DBA/2J animals, both would have been considered as equal in the human glaucoma datasets.

### *Spink2* Function in Retinal Ganglion Cell Apoptosis

Previous to this report, the study of *Spink2* has been limited to the examination of its function in mouse spermatogenesis [19]. Male mice with reduced *Spink2* expression levels exhibit abnormal spermatogenesis and reduced sperm count, which was associated with an increase in germ cell apoptosis. These phenotypic effects have been attributed to an increase in serine-protease activity, which may play an as yet undescribed role in regulating apoptosis in male germ cells [19].

There are 10 known isoforms of the *SPINK* gene family in humans. Mutations in *SPINK1* (*Spink3* in mice) have been genetically linked to pancreatitis in humans. *Spink3* knock-out mice mimic human pancreatitis, with a phenotype showing uncontrolled activation of autophagy in acinar cells [26], [27]. Similarly, knock-down of expression of the *SPINK* homologous gene, *Kazal1*, in the Cnidarian Hydra leads to a similar deregulation of autophagy in endodermal digestive cells [28], [29]. Whether or not the ability of Kazal domain-containing proteins to regulate autophagy is linked to their ability to inhibit proteases is unknown.

Mutations in the human *SPINK5* (*LEKTI*) gene lead to Netherton syndrome [30] characterized by abnormal desquamation and impaired cornification. These defects have been associated with hyperactivity of dermal proteases leading to increased degradation of the desmosomal junction protein desmoglein 1 [31].

Of the actions attributed to SPINK isoforms, a role in autophagy provides the most enticing mechanism for the effect of *Spink2* on increasing the susceptibility of retinal ganglion cells to optic nerve damage. Autophagy, itself, has been implicated both positively and negatively in neuronal damage. Neurons with a normal autophagic response, however, are much more capable of resisting damaging stresses [32], [33]. Using a similar experimental paradigm to our optic nerve crush protocol, Rodriguez-Muela and colleagues [34], recently demonstrated a protective role for autophagy in mouse ganglion cells after optic nerve axotomy. Axotomy induces an autophagic flux in ganglion cells, while pre-induction of autophagy in mice with rapamycin is able to increase the resistance of these cells to optic nerve damage. Finally, *Atg4b*-deficient mice, which have an impaired autophagic flux, exhibit increased susceptibility to axotomy.

Genes that affect the autophagic flux may also be implicated in increased ganglion cell susceptibility. Both optineurin (*OPTN*) and TANK-binding protein 1 (*TBK1*) have been genetically linked to patients with normal tension glaucoma [35], [36], [37], [38], [39]. The OPTN protein functions by first binding to protein aggregates. TBK1, which co-localizes with OPTN and these aggregates, phosphorylates OPTN and enhances its interaction with *Atg8* proteins that are essential for recruitment of the autophagy machinery and maturation of autophagosomes [40], [41]. Protein aggregates are subsequently cleared through an ubiquitin-independent proteolysis pathway. Importantly, mutations in *OPTN*, and a gene duplication of *TBK1* leading to increased expression, have been linked to normal tension glaucoma. Given the hypothesis that any level of intraocular pressure exposes ganglion cells to strain on the optic nerve head [42], we can speculate that an alteration in the autophagic flux of ganglion cells decreases their threshold to tolerate this damaging influence.

The differential effect of *Spink2* expression on increased susceptibility in BALB/cByJ mice could be the result of higher expression and/or the T19S polymorphism. The level of expression could be especially relevant in dying cells, which appear to exhibit the most intense staining for SPINK2 protein (Figure 7). In D407 cells, both variants were likely overexpressed compared to the levels of this protein in normal RGCs, although it is not clear how this level of expression compared with the levels present in the dying cells. Nevertheless, there was still a differential effect caused by the polymorphism, arguing that the principal difference between strains is correlated with SPINK2 protein function. This amino acid change would normally be considered conservative, and therefore not be expected to alter protein structure and function significantly. Additionally, this change occurs well in front of the Kazal protease inhibitory domain, which has been shown, in vitro, to inhibit trypsin activity [19], [20]. Consequently, it seems unlikely that the differential effect of the *Spink2* variants occurs by modifying protease activity in cells. A potential effect of this amino acid change may also be in altering any post-translational modifications of the SPINK2 protein. A search of the PhosphositePlus database, however, does not reveal any known modifications to this protein in this region. This may be a function of the relatively limited study of this protein to date.

### Conclusions

The *Rgcs1* QTL, possibly reflected by variants in the *Spink2* gene, modifies susceptibility of mouse retinal ganglion cells to both acute and chronic damage to the optic nerve. This accounts for an 11% influence on the cell death phenotype. In a complex genetic disease, such as glaucoma, this allele may work in combination with other genes to affect the susceptibility and severity of disease progression in humans with glaucoma. How *Spink2* variants influence the cell death process is unkown, but regulation of autophagy appears to be a likely candidate process.

## Methods

### Animals, Optic Nerve Crush Surgery, and Chronic Glaucoma

Adult mice were used for this study and handled in accordance with the Association for Research in Vision and Ophthalmology statement for the use of animals in research. All experimental protocols were approved by the Animal Care and Use Committee of the University of Wisconsin. Mice were housed in microisolator cages and kept on a 12 hr light/dark cycle and maintained on a 4% fat diet (8604 M/R, Harland Teklad, Madison, WI). Purebred parental strain mice were purchased directly from the Jackson Laboratories (Bar Harbor, ME). DBA/2J.BALB*^Rgcs1^* substrain mice were bred by successive crosses of pure bred DBA/2J animals with backcrossed mice carrying the BALB/cByJ *Rgcs1* QTL. Crosses were randomly made to male or female DBA/2J pure bred mice. For initial crosses, the size of the QTL was defined by microsatellite markers D5Mit254 and D5Mit338. As the interval of the *Rgcs1* locus was refined, subsequent backcrossed pups were selected using SNPs within 76.7 and 77.7 Mbp of chromosome 5. The substrain was considered congenic after 10 generations.

For optic nerve crush experiments, we maintained the age/crush paradigm originally set out in Li et al [10]. Briefly, purchased or bred animals were aged to 8 weeks before undergoing optic nerve crush surgery as described previously [10], [11], [43]. After two weeks, animals were euthanized and the retinas whole-mounted for surface staining of the cells in the ganglion cell layer. Cell loss was determined for each mouse by comparing neuronal cell density in the superior retina of the crush eye with the same region of the non-surgical eye. This method of quantification proved robust enough to consistently demonstrate inbred strain differences, including phenotype changes associated with reciprocal backcrossings and the original identification of the *Rgcs1* QTL. We did not deviate from the age restriction of 8 weeks for any experiments utilizing the crush protocol.

For chronic glaucoma, we took advantage of the fact that the resistant DBA/2J inbred strain is also a commonly used model for secondary glaucoma. It is important to note that disease onset and progression is well after the 8 week age time point, so disease was not expected to confound results of the crush experiments in this strain. Briefly, DBA/2J mice exhibit anterior segment dysgenesis caused by mutations in the *Gpnmb* and *Tyrp1* genes. This leads to iris stromal atrophy and pigment dispersion and finally senechae formation in the trabecular meshwork. Generally a population of DBA/2J animals will develop increased IOP at around 6 months of age, followed by optic nerve and retinal degeneration at around 8–12 months of age [14], [15]. For glaucoma studies, we examined cohorts of DBA/2J (DD), and DBA/2J.BALB*^Rgcs1^* substrain mice carrying 1 or 2 BALB/cByJ alleles (CC and CD, respectively) aged to 3 months and 10 months. IOP measurements were made by applanation tonometry (Tono-Lab, Colonial Medical Supply, Franconia, NH), while mice were anesthetized with ketamine (6 mg/ml) and xylazine (0.4 mg/ml). Measurements were taken 5–10 minutes after anesthesia, when normalized pressures under this anesthetic are most similar to IOPs in awake mice [44]. Glaucomatous damage was assessed as a function of optic nerve degeneration and cell loss in the retina. Optic nerve degeneration was evaluated from sections stained for βIII-tubulin [45] and scored as having mild, moderate, or severe damage using the system outlined in Libby et al [14]. Cell density in the retina was evaluated from retina whole mounts as described for optic nerve crush experiments. Numbers of samples used for glaucoma studies included 20 and 163 DD, 28 and 90 DC, and 15 and 45 CC eyes (3 and 10 months, respectively).

### SNP Mapping

The *Rgcs1* region was originally identified using microsatellite markers to screen a mapping population of 100 F2 mice selected from the extreme phenotypes of a larger population of 196 animals [11]. For SNP mapping, the entire F2 population of mice was used, including an additional 56 individuals for a total of 252 mice. In the first screen, a total of 37 SNPs located between 34 and 59 cM (the original region defining the *Rgcs1* allele [11]). A second screening using 7 further SNPs located between 76.7 and 77.7 Mbp. All SNP assays were conducted using TaqMan SNP assay technology and run on an ABI 7300 Real-Time qPCR machine (Applied Biosystems/Life Technologies, Grand Island, NY). Analysis was done using scanone in R/QTL with the EM method [46] using 1000 permutations.

### Reverse Transcriptase-PCR

For tissue distribution of *Spink2* mRNA, total RNA was isolated using an acid phenol extraction method [47] from the following BALB/cByJ mouse whole tissue lysates; brain, retina, lung, heart, liver, spleen, and testes. First strand cDNA was synthesized from 2 μg of total RNA using oligo-d(T) as a primer, with or without reverse transcriptase. RT-PCR was performed on approximately 200 pg of cDNA from each tissue. Primers used to amplify *Spink2* exons 1–4 (464 bp) were forward primer 5′-ACCACTGCTCTGTTCGCTGCAC and reverse primer 5′-CTGTCTTCCAGCCTCTACCC. Primers used to amplify *Spink2* exons 3–4 (242 bp) were forward primer 5′ TCAACCCTGTGTGCGGAACG and the same reverse primer. The PCR cycling parameters were: 1 cycle at 95°C for 5 min, 40 cycles of 95°C for 30 sec, 60°C for 30 sec, and 72°C for 1 min, and 72°C extension for 5 min. A cDNA amplimer of the *Rest* mRNA was used as a positive control and amplified with forward primer 5′- ACGCGAATGCGGACTCA and reverse primer 5′-CGCCTAGTCACACACGGGGC. PCR products from all primer sets were verified by sequencing. RT-PCR products were visualized on ethidium bromide stained 1% agarose gels.

### Quantitative Real-time PCR

Prior to qPCR analysis, routine RT-PCR was conducted using retinal and optic nerve material to determine if any of the genes in the 1 Mb region of the *Rgcs1* QTL were expressed in these tissues. Additionally, cDNA from brain, heart, lung, liver, and spleen tissues were also screened. Primers were designed for each putative expressed gene in this region. Primer design criteria were to create amplimers between 200–400 bp and span at least one intron. Total retinal or optic nerve RNA was extracted and used to make first strand cDNA with oligo-(dT) as a primer as described above. RT-PCR was performed using a temperature gradient between 50°C and 65°C on Mastercycler EP grandient thermocycler (Eppendorf, Hamburg, FRG). If the primer pair yielded a band of the expected size, it was isolated and cloned into pGEM-T cloning vector (Promega, Madison, WI). The identity of all bands was confirmed by sequencing. If the initial primer did not yield a band, two additional sets of primer pairs were designed for the target gene. If no PCR product was obtained in the 3 independent attempts, that target gene was not investigated further. Primers for all genes found to be expressed in the retina and/or optic nerve are shown in Table S1.

For qPCR experiments, DBA/2J or BABL/cByJ mice were subjected to optic nerve crush and euthanized 7 days later. Retinas and optic nerves from control and experimental eyes were pooled from 5 mice of each strain and frozen for RNA extraction as described above. For cDNA first strand synthesis, 3 μg of total RNA from either the retina or optic nerves were used with oligo-dT. From this material, approximately 1 ng of cDNA was input into each qPCR reaction, and each assay was run in triplicate for any given qPCR run. Reactions were run on an ABI 7300 Real-Time qPCR machine (Applied Biosystems/Life Technologies). Only genes in which retinal/optic nerve expression was confirmed were assayed. Quantification of transcript abundance was made using the absolute method against a standard curve of transcript copy number included directly on the array. In some experiments, the standard curve was an increasing copy number of the cloned target cDNA sequence. In other experiments, the standard curve was for a cDNA for the *S16* ribosomal protein gene, with no difference in result. *S16* cDNA and *Gapdh* cDNA were also included as loading control genes in each array.

### Copy Number Variant Analysis

Copy number analysis of the *Spink2* gene was carried out using the TaqMan Copy Number Assay (Applied Biosystems/Life Technologies) select for exon 4 of this gene (assay ID Mm00565370_cn), with the *Tfrc* gene as a 2-allele specific internal reference control.

### Plasmid Constructs

To create a construct containing a *Spink2*-BFP fusion protein, a 275 bp fragment was amplified from a construct containing either the DBA/2J or BALB/cByJ *Spink2* coding region using forward primer 5′-GACTGCGATCTCGAGATGCTGAGACTG and reverse primer 5′-TGCAAGTGCGAATTCTGCATGGCTCGTC. This fragment included the start codon for *Spink2*, omitted the stop codon and was engineered to have a 5′ XhoI site and 3′ EcoRI site. This PCR fragment was ligated into the pGem-T vector (Promega). Both pGemT-*Spink2* and destination vector pTagBFP-N (Evrogen, Moscow, Russia) were digested with restriction enzymes XhoI and EcoRI (Promega). The digested fragment and vector were gel purified by phenol/chloroform extraction. The *Spink2* fragment was ligated to pTagBFP-N in a 3∶1 insert:vector ratio using T4 DNA ligase (Promega). This placed BFP in frame with the *Spink2* coding region at the C-terminus of the SPINK2 protein. The complete pTagBFP-SPINK2 was confirmed by PCR and sequence analysis.

Construction of the peGFP-BAX plasmid was previously described in Semaan et al [48].

### BAX Aggregation Assay

D407 tissue culture cells (immortalized human retinal pigment epithelium) were cultured in DMEM containing 4.5 g/L glucose with L-glutamine (Corning Cellgro, Mediatech, Inc, Manassas, VA) supplemented with 3% FBS (Atlanta Biologicals, Norcross, GA), 1% penicillin/streptomycin and 0.0025% Amphotericin B. A Nucleofector-2b device was used for all cell transfections (Lonza, Allendale, NJ*)*. Each nucleofected reaction contained 1×10^6^ cells and was transfected with 1 μg peGFP-BAX as a control or 1 μg peGFP-BAX and 1 μg pTagBFP-SPINK2. Cells were live-imaged using an Andor Revolution XD confocal microscope (Andor Technology, Belfast, UK). At 24 hours post-nucleofection, cells were challenged with 1 μM staurosporine (STS) to induce cell death. Images were collected at time zero, 1 hour and 2 hours following staursporine addition. Commitment to apoptosis was monitored as a function of BAX aggregation. Upon a cell death stimulus, cytosolic BAX will translocate to, and aggregate at the mitochondrial outer membrane (MOM) [48], [49]. BAX aggregation leads to permeabilization of the MOM, release of cytochrome C, and finally, activation of the caspase cascade. The BAX translocation event is essentially the cell’s committed step to carry out the apoptotic pathway [50]. Using fluorescent tagged GFP-BAX, the translocation event is visualized as the transition from diffuse cytosolic staining to punctate GFP co-localizing with the mitochondria. The percentage of punctate cells in a given field was obtained by counting the number of cells containing BAX puncta divided by the total number of peGFP-BAX transfected or peGFP-BAX pTagBFP-SPINK2 co-transfected cells in the field.

### Caspase Assays

D407 cells were plated at a density of 1.5×10^4^ cells/well in a 96-well plate. Cells were transfected 24 hrs after plating using Transfast reagent (Promega) with a 3∶1 (transfection reagent: DNA) ratio. Plasmid DNAs peGFP, or pTarget-BALB/CByJ *Spink2* were used at a concentration of 100 ng/well. After 24 hrs, cells were treated with either 1 μM STS or DMSO for timepoints 0, 4, 8, 19 and 24 hours. STS treatments ended simultaneously, at which time, cells were incubated with Caspase 3/7 reagent from the APO-ONE Caspase 3/7 Assay *(*Promega) for 4 hours. Fluorescence was measured using a Tecan Safire^2^ Microplate Reader (Tecan US, Inc, Durham, NC).

### Immunolabeling

For sections, mice were euthanized at 5 and 7 days after optic nerve crush. Eyes were enucleated and fixed for 1 hr at 22°C in 4% paraformaldehyde in phosphate buffered saline (PBS, 150 mM NaCl, 100 mM Phosphate Buffer, pH 7.2). Eye cups containing the retina, but lacking the anterior chamber and lens, were post-fixed over night in 0.4% paraformaldehyde in PBS, before being equilibrated in PBS containing 30% sucrose. They were then embedded in optimal cutting temperature compound, (OCT, Thermo Fisher Scientific, Waltham, MA) and 5 μm sections involving the axis of the optic nerve were affixed to glass Plus slides (Thermo Fisher Scientific). Sections were blocked in PBS containing 4% bovine serum albumin (BSA) for 4 hrs at 4°C and then overlaid in PBS with 4% BSA containing a 1∶100 dilution of a polyclonal antibody against mouse SPINK2. Three different primary antibodies were tested, including a goat polyclonal against the C-terminus region (antibody sc-165566, Santa Cruz Biotechnology, Dallas, TX), a rabbit polyclonal against the C-terminus region (antibody 6667, ProSci Inc., Poway, CA), and a rabbit polyclonal made against a mouse SPINK2 fusion protein (a kind gift from Dr. Chunghee Cho, see reference 18). Sections were incubated at 4°C from overnight to 2 days, after which they were washed and incubated over night at 4°C with either a rabbit anti-goat IgG conjugated to Texas Red, or a goat anti-rabbit IgG conjugated to Texas Red, (1∶1000 dilution for both secondaries, Jackson ImmunoResearch, West Grove, PA). Sections were then washed and counterstained for 10 minutes with PBS containing 300 ng/mL 4′, 6′-diamindino-2-phenylindole (DAPI, Thermo Fisher Scientific) and coverslipped. Sections were viewed under a Zeiss Axioplan 2 compound fluorescent microscope (Zeiss Microimaging, Thornwood, NY) at 400× magnification. Immunolabeling controls included sections stained with only secondary antibodies, or sections stained with primary antibodies (Santa Cruz and ProSci antibodies only) after competition with a peptide corresponding to 16 amino acids at the C-terminus of human SPINK2 (ProSci, Inc). Supplemental Figure S4 shows minimal immunoreactivity of both the secondary and competed primary antibodies.

For retinal whole mounts, fixed eye cups were incubated for 4 hrs at 22°C in PBS containing 0.5% Triton-X100 and then blocked over night at 4°C in the same buffer containing 2% donkey serum (Jackson ImmunoResearch). Block was then removed and replaced with fresh blocking buffer containing 1∶100 dilution of the SPINK2 antibody (goat polyclonal from Santa Cruz or rabbit polyclonal from Dr. Cho) and 1∶100 dilution of an antibody against BRN3A (mouse monoclonal MAB1585, EMD Millipore, Billerica, MA) and incubated for 48 hrs at 4°C. After incubation the eye cups were incubated over night at 4°C in blocking buffer containing 1∶1000 dilutions each of either a donkey anti-goat IgG, or a goat anti-rabbit IgG, conjugated to fluorescein isothiocyanate (Jackson ImmunoResearch), and donkey anti-mouse IgG conjugated to Alexa 594 (Molecular Probes, Life Technologies). Retinas were then removed from the eye cups, flat mounted on glass Plus slides, and coverslipped. Images were obtained using an Andor Revolution XD confocal microscope.

For western blotting, retinal homogenates were prepared in sample buffer (62.5 mM Tris, pH 6.8, 2% SDS, 25% glycerol) and sonicated. Protein concentrations were determined using a Pierce bicinchoninic acid staining kit (Thermo Fisher Scientific). Equal loads of protein between 15 and 100 μg were then separated on 12% SDS-polyacrylamide gels and blotted to Immobilon-FL membrane (EMD Millipore). Membranes were probed with a 1∶100 dilution of either the SPINK2 antibody (Santa Cruz), or an antibody against β-ACTIN. After washing, they were incubated in appropriate IR Dye secondary antibodies (LI-COR Biosciences, Lincoln, NE) and screened on a LI-COR Odyssey CLx infrared imagining system. Protein bands were quantified using the LI-COR Image studio software.

### Statistical Analyses

The following statistical tests were used for this study. For cell loss studies involving a change in cell density in the ganglion cell layer data was examined by Student’s t-test when comparing two means and 1-way ANOVA when comparing multiple groups. Individual means were also tested by t-test. IOP data among groups were tested by ANOVA. Optic nerve damage scores were examined by Chi-square analysis using the frequency of damage scores in DBA/2J wild type mice as the expected frequencies, compared to DBA/2J.BALB*^Rgcs1^* substrain mice. Data collected using qPCR were assessed by t-test. Group sizes (n) are indicated in the respective descriptions in the methods. BAX aggregation studies were analyzed from a minimum of 46 microscopic fields (1 hr time point) or 32 microscopic fields (1.5 hr time point).pooled from 3–5 independent experiments. Statistical analyses was conducted using a general linear mixed model (using package Ime4 in R) to first assess that there is no treatment group by time interaction (P = 0.19), and then to assess the additive effects of treatment groups and of time points.

### Web Resources

Mouse Genome Informatics (www.informatics.jax.org)

Wellcome Trust Sanger Institute (www.sanger.ac.uk)

ENSEMBL (www.ensembl.org)

R/qtl (www.Rqtl.org)

Phosphosite Plus (www.phosphosite.org)

## Supporting Information

Figure S1 **A scatter plot of cell death phenotype as a function of distribution of SNP rs13478335 in the F2 mapping population.** Mice carrying the DBA/2J allele (D) exhibit the resistant phenotype, while mice homozygous for the BALB/cByJ allele (C) show the susceptible phenotype (P<0.001). The distribution of phenotypes indicates that the D allele is dominant. The mean ± SEM is indicated for each population.
(TIF)

Figure S2 **Quantitative PCR of mRNAs of genes in the** ***Rgcs1*** **QTL in the optic nerve after crush.** The majority of genes showed no substantial changes (i.e., more than a doubling) in transcript abundance after optic nerve damage, that was consistent in both stains. The exception was *Cep135*, which shows a significant increase in both strains (P<0.03).
(TIF)

Figure S3 **Retinal** ***Spink2*** **changes after optic nerve crush.** (A) Transcript abundance for *Spink2* mRNA between DBA/2J and BALB/cByJ mice. Quantitative PCR data showing absolute transcript abundance for *Spink2* mRNA in DBA/2J and BALB/cByJ retinas before and 7 days after optic nerve crush. Both strains show an increase in *Spink2* mRNA after crush (*P = 0.02, **P<0.01), but endogenous levels are higher in BALB/cByJ mice (P = 0.01). (B,C) Quantification of retinal SPINK2 protein in DBA/2J and DBA/2J.BALB*^Rgcs1^* substrain mice. Protein levels in the gel shown in (C) are indicated. In separate experiments, increases in SPINK2 ranged from 25–300%. SPINK2 levels are consistently higher in mice carrying the BALB/cByJ allele.
(TIF)

Figure S4 **Control panels of retinal sections for rabbit and goat polyclonal antibodies.** Sections of retinas from control mice are shown. (A) A section stained with a goat anti-rabbit IgG conjugated to Texas Red (GAR). (B) A section stained with a rabbit polyclonal antibody against the C-terminus of SPINK2 (ProSci Inc.), after competition with a peptide made from 16 amino acids of the C-terminus of human SPINK2. Counterstained with the GAR secondary. (C) A section stained with the ProSci antibody without peptide competition (GAR secondary). This antibody often appears to stain Müller cell processes (arrow). (D) A section stained only with the rabbit anti-goat IgG conjugated to Texas Red (RAG). (E) A section stained with a goat polyclonal antibody against the C-terminus of SPINK2 (Santa Cruz Biotechnology), after peptide competition. Counterstained with the RAG secondary. (F) A section stained with the Santa Cruz antibody without peptide competition (RAG secondary). All sections are DAPI counterstained.
(TIF)

Figure S5 **Caspase 3/7 activity levels in D407 cells after staurosporine (STS) induction of apoptosis.** Graph showing the caspase activity in D407 cells as a function of time after (STS) addition. Cells transfected with a GFP expression plasmid exhibit caspase activity indistinguishable from non-transfected cells. Cells transfected with the BALB/cByJ variant of *Spink2* exhibit significantly more caspase activity (*P<0.02 at 8 hours). These cells were not transfected with the *Spink2*-Blue fluorescent protein fusion construct shown in Figure 8.
(TIF)

Table S1 **Primer sequences for qPCR studies of genes in the 1 Mb** ***Rgcs1*** **region.** Genes and primers shown were those that yielded successful amplification of a target cDNA in the retina and optic nerve. All primers listed were used for qPCR analysis of retinal and optic nerve tissues after optic nerve crush in DBA/2J and BALB/cByJ mice.
(DOCX)

## References

[1] Libby RT, Li Y, Savinova OV, Barter J, Smith RS, et al (2005) Susceptibility to neurodegeneration in glaucoma is modified by Bax gene dosage. PLoS Genet 1: 17–26. 16103918 10.1371/journal.pgen.0010004 PMC1183523

[2] Quigley HA, Nickells RW, Kerrigan LA, Pease ME, Thibault DJ, et al (1995) Retinal ganglion cell death in experimental glaucoma and after axotomy occurs by apoptosis. Invest Ophthalmol Vis Sci 36: 774–786. 7706025

[3] Wiggs JL (2007) Genetic etiologies of glaucoma. Arch Ophthalmol 125: 30–37. 17210849 10.1001/archopht.125.1.30

[4] Ramdas WD, van Koolwijk LME, Lemij HG, Pasutto F, Cree AJ, et al (2011) Common genetic variants associated with open-angle glaucoma. Hum Mol Genet 20: 2464–2471. 21427129 10.1093/hmg/ddr120

[5] Burdon KP, Macgregor S, Hewitt AW, Sharma S, Chidlow G, et al (2011) Genome-wide association study identifies susceptibility loci for open angle glaucoma at TMCO1 and CDKN2B-AS1. Nat Genet 43: 574–580. 21532571 10.1038/ng.824

[6] Wiggs JL, Yaspan BL, Hauser MA, Kang JH, Allingham RR, et al (2012) Common variants at 9p21 and 8q22 are associated with increased susceptibility to optic nerve degeneration in glaucoma. PLoS Genet 8: e1002654. 22570617 10.1371/journal.pgen.1002654 PMC3343074

[7] Fingert JH (2011) Primary open-angle glaucoma genes. Eye 25. 10.1038/eye.2011.97 PMC3171270 21562585

[8] Howell GR, Libby RT, Jakobs TC, Smith RS, Phalan FC, et al (2007) Axons of retinal ganglion cells are insulted in the optic nerve early in DBA/2J glaucoma. J Cell Biol 179: 1523–1537. 18158332 10.1083/jcb.200706181 PMC2373494

[9] Nickells RW, Howell GR, Soto I, John SWM (2012) Under pressure: cellular and molecular responses during glaucoma, a common neurodegeneration with axonopathy. Ann Rev Neurosci 35: 153–179. 22524788 10.1146/annurev.neuro.051508.135728

[10] Li Y, Semaan SJ, Schlamp CL, Nickells RW (2007) Dominant inheritance of retinal ganglion cell resistance to optic nerve crush in mice. BMC Neurosci 8: 19. 17338819 10.1186/1471-2202-8-19 PMC1831479

[11] Dietz JA, Li Y, Chung LM, Yandell BS, Schlamp CL, et al (2008) *Rgcs1*, a dominant QTL that affects retinal ganglion cell death after optic nerve crush in mice. BMC Neurosci 9: 74. 18671875 10.1186/1471-2202-9-74 PMC2518923

[12] John SWM, Smith RS, Savinova OV, Hawes NL, Chang B, et al (1998) Essential iris atrophy, pigment dispersion, and glaucoma in DBA/2J mice. Invest Ophthalmol Vis Sci 39: 951–962. 9579474

[13] Chang B, Smith RS, Hawes NL, Anderson MG, Zabaleta A, et al (1999) Interacting loci cause severe iris atrophy and glaucoma in DBA/2J mice. Nature Genet 21: 405–409. 10192392 10.1038/7741

[14] Libby RT, Anderson MG, Pang I-H, Robinson ZH, Savinova OV, et al (2005) Inherited glaucoma in DBA/2J mice: pertinent disease features for studying the neurodegeneration. Vis Neurosci 22: 637–648. 16332275 10.1017/S0952523805225130

[15] Schlamp CL, Li Y, Dietz JA, Janssen KT, Nickells RW (2006) Progressive ganglion cell loss and optic nerve degeneration in DBA/2J mice is variable and asymmetric. BMC Neurosci 7: 66. 17018142 10.1186/1471-2202-7-66 PMC1621073

[16] Anderson MG, Smith RS, Hawes NL, Zabaleta A, Chang B, et al (2002) Mutations in genes encoding melanosomal proteins cause pigmentary glaucoma in DBA/2J mice. Nat Genet 30: 81–85. 11743578 10.1038/ng794

[17] Ohta T, Essner R, Ryu H, Palazzo RE, Uetake Y, et al (2002) Characterization of Cep1135, a novel coiled-coil centrosomal protein involved in microtubule organization in mammalian cells. J Cell Biol 156: 87–99. 11781336 10.1083/jcb.200108088 PMC2173569

[18] Johnson EC, Jia L, Cepurna WA, Doser TA, Morrison JC (2007) Global changes in optic nerve head gene expression after exposure to elevated intraocular pressure in a rat glaucoma model. Invest Ophthalmol Vis Sci 48: 3161–3177. 17591886 10.1167/iovs.06-1282 PMC1950563

[19] Lee B, Park I, Jin S, Choi H, Kwon JT, et al (2011) Impaired spermatogenesis and fertility in mice carrying a mutation in the Spink2 gene expressed predominantly in testes. J Biol Chem 286: 29108–29117. 21705336 10.1074/jbc.M111.244905 PMC3190718

[20] Chen T, Lee TR, Liang WG, Chang WSW, Lyu PC (2009) Identification of trypsin-inhibitory site and structure determination of human SPINK2 serine proteinase inhibitor. Prot Struct Func Bioinformatics 77: 209–219. 10.1002/prot.22432 19422058

[21] Agam A, Yalcin B, Bhomra A, Cubin M, Webber C, et al (2010) Elusive copy number variation in the mouse genome. PLoS One 5: e12839. 20877625 10.1371/journal.pone.0012839 PMC2943477

[22] Nadal-Nicolas FM, Jimenez-Lopez M, Sobrado-Calvo P, Nieto-Lopez L, Canovas-Martinez I, et al (2009) Brn3a as a marker of retinal ganglion cells: qualitative and quantitative time course studies in naive and optic nerve-injured retinas. Invest Ophthalmol Vis Sci 50: 3860–3868. 19264888 10.1167/iovs.08-3267

[23] Schlamp CL, Montgomery AD, Mac Nair CE, Schuart C, Willmer DJ, et al (2013) Evaluation of the percentage of ganglion cells in the ganglion cell layer of the rodent retina. Mol Vis 19: 1387–1396. 23825918 PMC3695759

[24] Weishaupt JH, Klocker N, Bahr M (2005) Axotomy-induced early down-regulation of POU-IV class transcription factors Brn-3a and Brn-3b in retinal ganglion cells. J Mol Neurosci 26: 17–25. 15968082 10.1385/JMN:26:1:017

[25] Pelzel HR, Schlamp CL, Nickells RW (2010) Histone H4 deacetylation plays a critical role in early gene silencing during neuronal apoptosis. BMC Neurosci 11: 62. 20504333 10.1186/1471-2202-11-62 PMC2886060

[26] Ohmuraya M, Sugano A, Hirota M, Takaoka Y, Yamamura K (2012) Role of intrapancreatic SPINK1/Spink3 expression in the development of pancreatitis. Front Physiol 3: 126. 22586407 10.3389/fphys.2012.00126 PMC3345944

[27] Ohmuraya M, Yamamura K (2011) The roles of serine protease inhibitor Kazal type 1 (SPINK1) in pancreatic diseases. Exp Anim 60: 433–444. 22041280 10.1538/expanim.60.433

[28] Chera S, de Rosa R, Miljkovic-Licina M, Dobretz K, Ghila L, et al (2006) Silencing of the hydra serine protease inhibitor Kazal1 gene mimics the human SPINK1 pancreatic phenotype. J Cell Sci 119: 846–857. 16478786 10.1242/jcs.02807

[29] Chera S, Buzgariu W, Ghila L, Galliot B (2009) Autophagy in Hydra: a response to starvation and stress in early animal evolution. Biochim Biophys Acta 1793: 1432–1443. 19362111 10.1016/j.bbamcr.2009.03.010

[30] Chavanas S, Bodemer C, Rochat A, Hamel-Teillac D, Ali M, et al (2000) Mutations in SPINK5, encoding a serine protease inhibitor, cause Netherton syndrome. Nat Genet 25: 141–142. 10835624 10.1038/75977

[31] Descargues P, Deraison C, Bonnart C, Kreft M, Kishibe M, et al (2005) Spink5-deficient mice mimic Netherton syndrome through degradation of desmoglein 1 by epidermal protease hyperactivity. Nat Genet 37: 56–65. 15619623 10.1038/ng1493

[32] Nedelsky NB, Todd PK, Taylor JP (2008) Autophagy and the ubiquitin-proteosome system: Collaborators in neuroprotection. Biochim Biophys Acta 1782: 691–699. 18930136 10.1016/j.bbadis.2008.10.002 PMC2621359

[33] Sternberg C, Benchimol M, Linden R (2010) Caspase dependence of the death of neonatal retinal ganglion cells induced by axon damage and induction of autophagy as a survival mechanism. Braz J Med Biol Res 43: 950–956. 20802972 10.1590/s0100-879x2010007500082

[34] Rodriguez-Muela n, Germain F, Marino G, Fitze PS, Boya P (2012) Autophagy promotes survival of retinal ganglion cells after optic nerve axotomy in mice. Cell Death Differ 19: 162–169. 21701497 10.1038/cdd.2011.88 PMC3252838

[35] Alward WL, Kwon YH, Kawase K, Craig JE, Hayreh SS, et al (2003) Evaluation of optineurin sequence variations in 1,048 patients with open-angle glaucoma. Am J Ophthalmol 136: 904–910. 14597044 10.1016/s0002-9394(03)00577-4

[36] Leung YF, Fan BJ, Lam DS, Lee WS, Tam PO, et al (2003) Different optineurin mutation pattern in primary open-angle glaucoma. Invest Ophthalmol Vis Sci 44: 3880–3884. 12939304 10.1167/iovs.02-0693

[37] Rezaie T, Child A, Hitchings R, Brice G, Miller L, et al (2002) Adult-onset primary open-angle glaucoma caused by mutations in optineurin. Science 295: 1077–1079. 11834836 10.1126/science.1066901

[38] Fingert JH, Robin AL, Stone JL, Roos B, Davis LK, et al (2011) Copy number variations on chromosome 12q14 in patients with normal tension glaucoma. Hum Mol Genet 20: 2482–2494. 21447600 10.1093/hmg/ddr123 PMC3098731

[39] Kawase K, Allingham RR, Meguro A, Mizuki N, Roos B, et al (2012) Confirmation of TBK1 duplication in normal tension glaucoma. Exp Eye Res 96: 178–180. 22306015 10.1016/j.exer.2011.12.021 PMC3296819

[40] Weidberg H, Elazar Z (2011) TBK1 mediates crosstalk between the innate immune response and autophagy. Sci Signal 4: pe39. 21868362 10.1126/scisignal.2002355

[41] Korac J, Schaeffer V, Kovacevic I, Clement AM, Jungblut B, et al (2013) Ubiquitin-independent function of optineurin in autophagic clearance of protein aggregates. J Cell Sci 126: 580–592. 23178947 10.1242/jcs.114926 PMC3654196

[42] Burgoyne CF (2011) A biomechanical paradigm for axonal insult within the optic nerve head in aging and glaucoma. Exp Eye Res 93: 120–132. 20849846 10.1016/j.exer.2010.09.005 PMC3128181

[43] Li Y, Schlamp CL, Nickells RW (1999) Experimental induction of retinal ganglion cell death in adult mice. Invest Ophthalmol Vis Sci 40: 1004–1008. 10102300

[44] Ding C, Wang P, Tian N (2011) Effect of general anesthetics on IOP in elevated IOP mouse model. Exp Eye Res 92: 512–520. 21457709 10.1016/j.exer.2011.03.016 PMC3116023

[45] Pelzel HR, Schlamp CL, Waclawski M, Shaw MK, Nickells RW (2012) Silencing of Fem1c^R3^ gene expression in the DBA/2J mouse precedes retinal ganglion cell death and is associated with Histone Deacetylase activity. Invest Ophthalmol Vis Sci 53: 1428–1435. 22297488 10.1167/iovs.11-8872 PMC3339913

[46] Lander ES, Botstein D (1989) Mapping Mendelian factors underlying quantitative traits using RFLP linkage maps. Genetics 121: 185–199. 2563713 10.1093/genetics/121.1.185 PMC1203601

[47] Schlamp CL, Nickells RW (1996) Light and dark cause a shift in the spatial expression of a neuropeptide processing enzyme in the rat retina. J Neurosci 16: 2164–2171. 8601797 10.1523/JNEUROSCI.16-07-02164.1996 PMC6578520

[48] Semaan SJ, Nickells RW (2010) The apoptotic response in HCT116^BAX−/−^ cancer cells becomes rapidly saturated with increasing expression of a GFP-BAX fusion protein. BMC Cancer 10: 554. 20942963 10.1186/1471-2407-10-554 PMC2964639

[49] Wolter KG, Hsu YT, Smith CL, Nechushtan A, Xi XG, et al (1997) Movement of bax from the cytosol to mitochondria during apoptosis. J Cell Biol 139: 1281–1292. 9382873 10.1083/jcb.139.5.1281 PMC2140220

[50] Chang LK, Putcha GV, Deshmukh M, Johnson Jr EM (2002) Mitochondrial involvement in the point of no return in neuronal apoptosis. Biochimie 84: 223–231. 12022953 10.1016/s0300-9084(02)01372-x

