# Supplementary figures and images for: Spink2 Modulates Apoptotic Susceptibility and Is a Candidate Gene in the Rgcs1 QTL That Affects Retinal Ganglion Cell Death after Optic Nerve Damage
Source: PLoS One. 2014 Apr 3;9(4):e93564. doi: 10.1371/journal.pone.0093564 (PMC3974755; doi:10.1371/journal.pone.0093564)

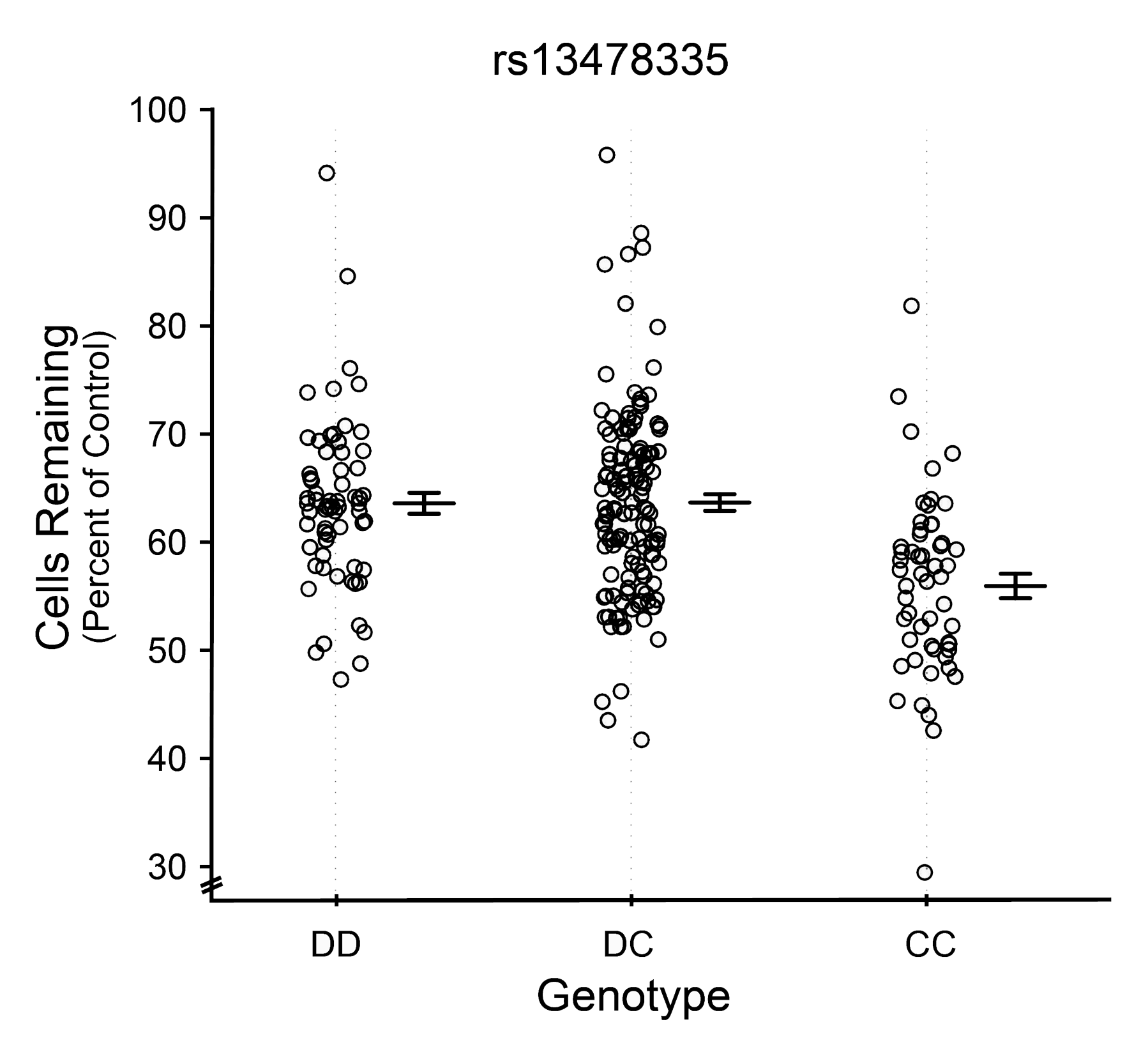

Supplement: Figure S1 — A scatter plot of cell death phenotype as a function of distribution of SNP rs13478335 in the F2 mapping population. Mice carrying the DBA/2J allele (D) exhibit the resistant phenotype, while mice homozygous for the BALB/cByJ allele (C) show the susceptible phenotype (P<0.001). The distribution of phenotypes indicates that the D allele is dominant. The mean ± SEM is indicated for each population. (TIF) [file pone.0093564.s001.tif]

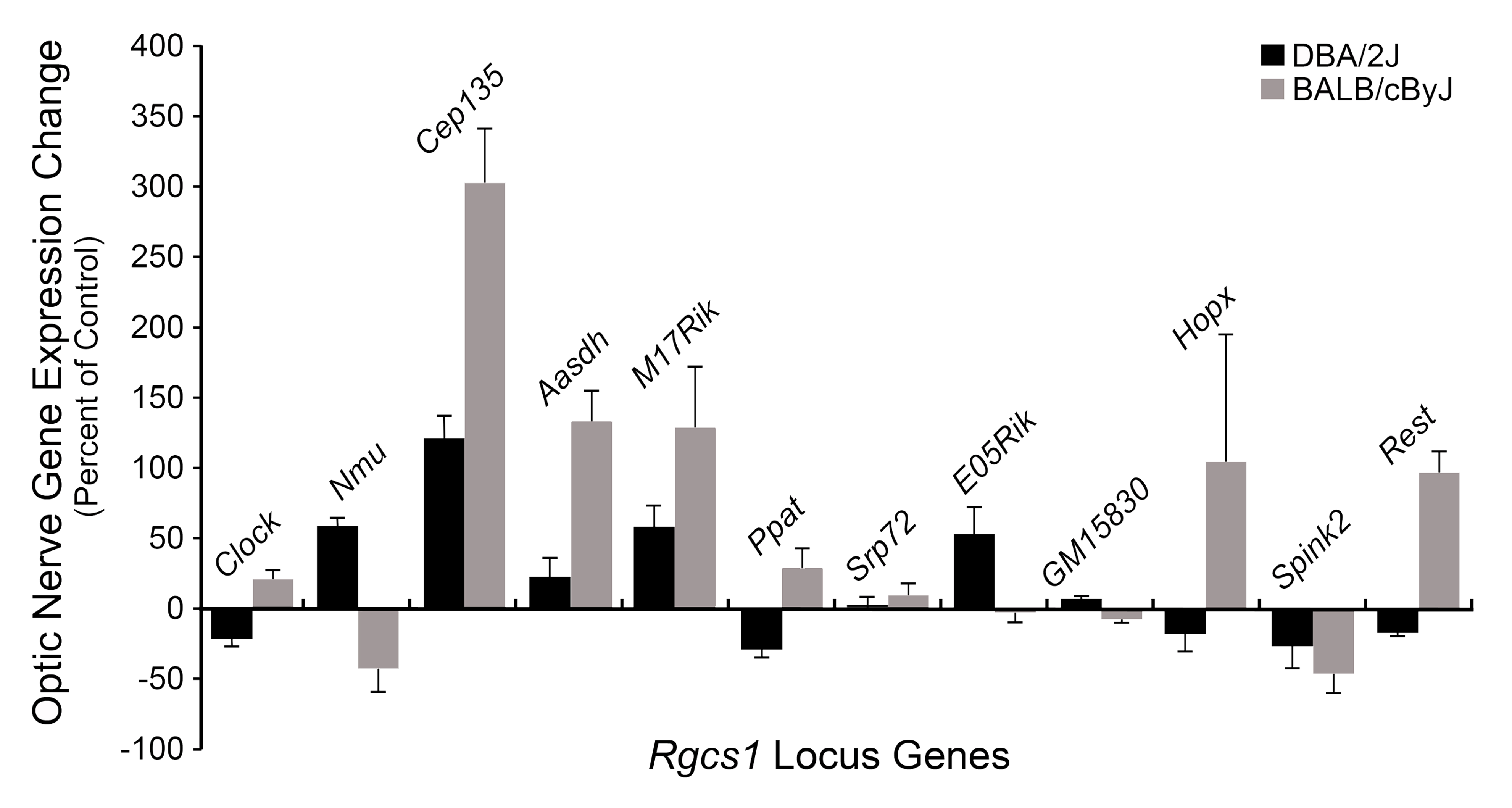

Supplement: Figure S2 — Quantitative PCR of mRNAs of genes in the Rgcs1 QTL in the optic nerve after crush. The majority of genes showed no substantial changes (i.e., more than a doubling) in transcript abundance after optic nerve damage, that was consistent in both stains. The exception was Cep135, which shows a significant increase in both strains (P<0.03). (TIF) [file pone.0093564.s002.tif]

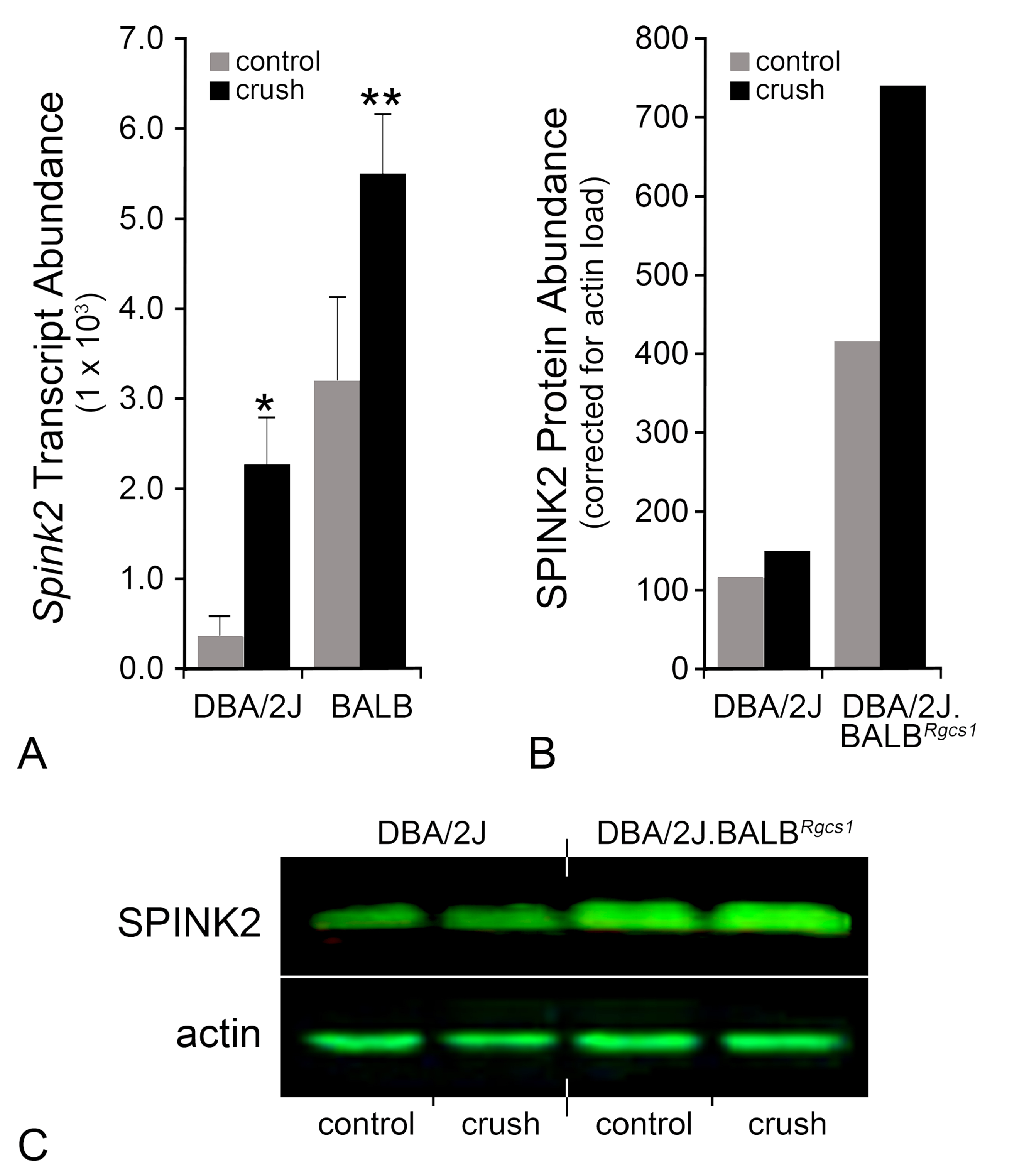

Supplement: Figure S3 — Retinal Spink2 changes after optic nerve crush. (A) Transcript abundance for Spink2 mRNA between DBA/2J and BALB/cByJ mice. Quantitative PCR data showing absolute transcript abundance for Spink2 mRNA in DBA/2J and BALB/cByJ retinas before and 7 days after optic nerve crush. Both strains show an increase in Spink2 mRNA after crush (*P = 0.02, **P<0.01), but endogenous levels are higher in BALB/cByJ mice (P = 0.01). (B,C) Quantification of retinal SPINK2 protein in DBA/2J and DBA/2J.BALBRgcs1 substrain mice. Protein levels in the gel shown in (C) are indicated. In separate experiments, increases in SPINK2 ranged from 25–300%. SPINK2 levels are consistently higher in mice carrying the BALB/cByJ allele. (TIF) [file pone.0093564.s003.tif]

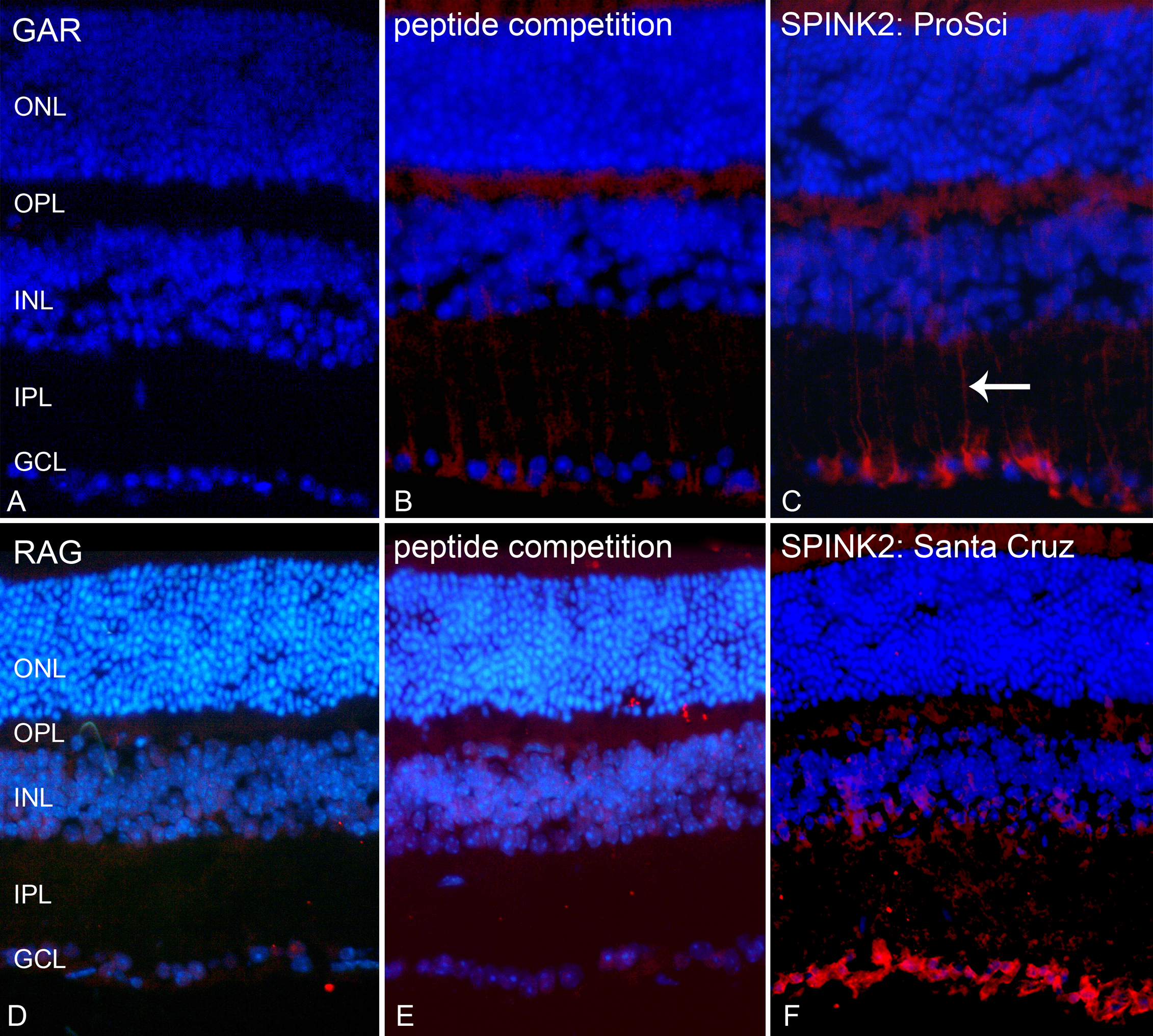

Supplement: Figure S4 — Control panels of retinal sections for rabbit and goat polyclonal antibodies. Sections of retinas from control mice are shown. (A) A section stained with a goat anti-rabbit IgG conjugated to Texas Red (GAR). (B) A section stained with a rabbit polyclonal antibody against the C-terminus of SPINK2 (ProSci Inc.), after competition with a peptide made from 16 amino acids of the C-terminus of human SPINK2. Counterstained with the GAR secondary. (C) A section stained with the ProSci antibody without peptide competition (GAR secondary). This antibody often appears to stain Müller cell processes (arrow). (D) A section stained only with the rabbit anti-goat IgG conjugated to Texas Red (RAG). (E) A section stained with a goat polyclonal antibody against the C-terminus of SPINK2 (Santa Cruz Biotechnology), after peptide competition. Counterstained with the RAG secondary. (F) A section stained with the Santa Cruz antibody without peptide competition (RAG secondary). All sections are DAPI counterstained. (TIF) [file pone.0093564.s004.tif]

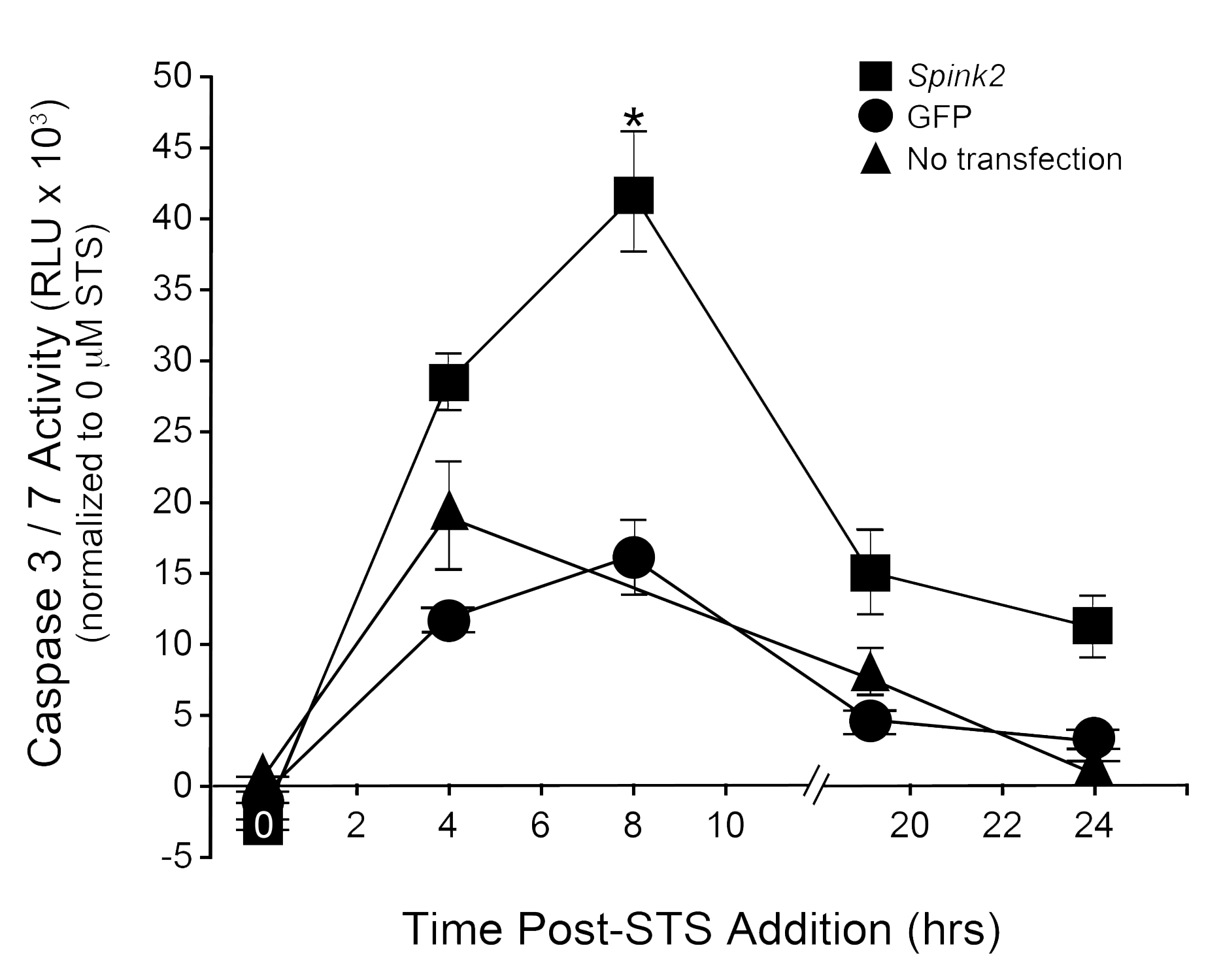

Supplement: Figure S5 — Caspase 3/7 activity levels in D407 cells after staurosporine (STS) induction of apoptosis. Graph showing the caspase activity in D407 cells as a function of time after (STS) addition. Cells transfected with a GFP expression plasmid exhibit caspase activity indistinguishable from non-transfected cells. Cells transfected with the BALB/cByJ variant of Spink2 exhibit significantly more caspase activity (*P<0.02 at 8 hours). These cells were not transfected with the Spink2-Blue fluorescent protein fusion construct shown in Figure 8. (TIF) [file pone.0093564.s005.tif]
