# Supplementary material for: Spink2 Modulates Apoptotic Susceptibility and Is a Candidate Gene in the Rgcs1 QTL That Affects Retinal Ganglion Cell Death after Optic Nerve Damage
Source: PLoS One. 2014 Apr 3;9(4):e93564. doi: 10.1371/journal.pone.0093564 (PMC3974755; doi:10.1371/journal.pone.0093564)
Supplement: Table S1 — Primer sequences for qPCR studies of genes in the 1 Mb Rgcs1 region. Genes and primers shown were those that yielded successful amplification of a target cDNA in the retina and optic nerve. All primers listed were used for qPCR analysis of retinal and optic nerve tissues after optic nerve crush in DBA/2J and BALB/cByJ mice. (DOCX) [file pone.0093564.s006.docx]

| **Gene** | **FWD Primer Sequence (5’ to 3’)** | **Exon** | **REV Primer Sequence (5’ to 3’)** | **Exon** | **Size (bp)** |
| --- | --- | --- | --- | --- | --- |
| *Clock* | ACA CAG AAG ACG GCC TTG CG | 3 | GAA GCA TAG ACC CCA GCT CC | 6 | 295 |
| *Nmu* | AGGGTGTGCCAATATCACCTCAAAGAT | 1/2 | CTTGTTGACCTCTTCCCGTTGCGTGGC | 8/9 | 398 |
| *Cep135* | TTG CAT TTA GGC GCC AGC GG | 4 | GAA CAA CCG CCA TCC AAC GC | 6 | 267 |
| *Aasdh* | AGT TGG TGC TTC GGA CTG CC | 4 | TCA ATG GGC GCA TAC GCT GC | 5 | 253 |
| *M17Rik* | ACT TCG TCC TCA CCA GTC CC | 3 | GCA GCA CTG TTG TCC AGA CG | 5 | 255 |
| *Ppat* | CTG AAG GAT GGG TGG TGT CC | 6 | CCA CGG GTG CTT CAA TTG CC | 8 | 269 |
| *Srp72* | AGA GGA GCT GAG GCA CAA GC | 12 | TGC TAT CCG ATG AGG GCA GG | 16 | 337 |
| *E05Rik* | GGA CAG GAG GCT GTT TTG GG | 3/4 | TGG GTT TGG CCA GCT TCT GG | 5 | 242 |
| *GM15830* | GGCTACCTACCTCACTGCGGTCT | 1 | GGC CAG GTC ACG CAT CAG CA | 2 | 338 |
| *Hopx* | TTC AGG TCC CTG CTT GAG CG | 1 | TTT CTG CGT CTG CTC CTC CG | 2/3 | 297 |
| *Spink2* | TCA ACC CTG TGT GCG GAA CG | 3 | CTG TCT TCC AGC CTC TAC CC | 4 | 242 |
| *Rest* | ACG CGA ATG CGG ACT CA | 2 | CGC CTA GTC ACA CAC GGG GC | 3 | 233 |
